# Subcellular Localization and Assembly Process of the Nisin Biosynthesis Machinery in Lactococcus lactis

**DOI:** 10.1128/mBio.02825-20

**Published:** 2020-11-10

**Authors:** Jingqi Chen, Auke J. van Heel, Oscar P. Kuipers

**Affiliations:** a Department of Molecular Genetics, Groningen Biomolecular Sciences and Biotechnology Institute, University of Groningen, Groningen, the Netherlands; University of Minnesota Medical School

**Keywords:** *Lactococcus lactis*, assembly, biosynthesis machinery, lantibiotics, subcellular localization

## Abstract

Nisin, a class I lantibiotic, is synthesized as a precursor peptide by a putative membrane-associated lanthionine synthetase complex consisting of the dehydratase NisB, the cyclase NisC, and the ABC transporter NisT. Here, we characterize the subcellular localization and the assembly process of the nisin biosynthesis machinery in Lactococcus lactis by mutational analyses and fluorescence microscopy. Precursor nisin, NisB, and NisC were found to be mainly localized at the cell poles, with a preference for the old poles. They were found to be colocalized at the same spots in these old pole regions, functioning as a nisin modification complex. In contrast, the transporter NisT was found to be distributed uniformly and circumferentially in the membrane. When nisin secretion was blocked by mutagenesis of NisT, the nisin biosynthesis machinery was also visualized directly at a polar position using fluorescence microscopy. The interactions between NisB and other components of the machinery were further studied *in vivo*, and therefore, the “order of assembly” of the complex was revealed, indicating that NisB directly or indirectly plays the role of a polar “recruiter” in the initial assembly process. Additionally, a potential domain that is located at the surface of the elimination domain of NisB was identified to be crucial for the polar localization of NisB. Based on these data, we propose a model wherein precursor nisin is first completely modified by the nisin biosynthesis machinery, preventing the premature secretion of partially modified peptides, and subsequently secreted by recruited NisT, preferentially at the old pole regions.

## Abstract (precis)

Nisin is the model peptide for LanBC-modified lantibiotics that are commonly modified and exported by a putative synthetase complex. Although the mechanism of maturation, transport, immunity, and regulation is relatively well understood, and structural information is available for some of the proteins involved (B. Li, J. P. J. Yu, J. S. Brunzelle, G. N. Moll, et al., Science 311:1464–1467, 2006, https://doi.org/10.1126/science.1121422; M. A. Ortega, Y. Hao, Q. Zhang, M. C. Walker, et al., Nature 517:509–512, 2015, https://doi.org/10.1038/nature13888; C. Hacker, N. A. Christ, E. Duchardt-Ferner, S. Korn, et al., J Biol Chem 290:28869–28886, 2015, https://doi.org/10.1074/jbc.M115.679969; Y. Y. Xu, X. Li, R. Q. Li, S. S. Li, et al., Acta Crystallogr D Biol Crystallogr 70:1499–1505, 2014, https://doi.org/10.1107/S1399004714004234), the subcellular localization and assembly process of the biosynthesis complex remain to be elucidated. In this study, we determined the spatial distribution of nisin synthesis-related enzymes and the transporter, revealing that the modification and secretion of the precursor nisin mainly occur at the old cell poles of L. lactis and that the transporter NisT is probably recruited later to this spot after the completion of the modification reactions by NisB and NisC. Fluorescently labeled nisin biosynthesis machinery was visualized directly by fluorescence microscopy. To our knowledge, this is the first study to provide direct evidence of the existence of such a complex *in vivo*. Importantly, the elucidation of the “order of assembly” of the complex will facilitate future endeavors in the investigation of the nisin secretion mechanism and even the isolation and structural characterization of the complete complex.

## INTRODUCTION

Lanthipeptides are ribosomally synthesized natural products posttranslationally modified by one or more enzymes (1). Lanthipeptides with antimicrobial activity (lantibiotics) are produced mainly by Gram-positive bacteria and act mostly against Gram-positive bacteria (2). The precursor peptide (LanA) of lanthipeptides is produced with an N-terminal leader peptide and a C-terminal core peptide (1). The leader peptide serves as a signal sequence and recognition site for the modification enzymes and export protein (3) and also keeps the modified peptide inactive (4). Posttranslational modification occurs within the core peptide but not in the leader peptide. Depending on the biosynthetic enzymes, lanthipeptides are mainly classified into four different classes (I to IV) (5). Class I lanthipeptides are dehydrated by a LanB enzyme and cyclized by a LanC enzyme, whereas in classes II, III, and IV, both reactions are conducted by a single bifunctional enzyme, referred to as LanM, LanKC, and LanL, respectively (6). Recently, a novel lanthipeptide, lexapeptide, was suggested to be one of the class V lanthipeptides considering the unique triprotein Lan synthetase composed of LxmK, LxmX, and LxmY (7).

**FIG 1:**
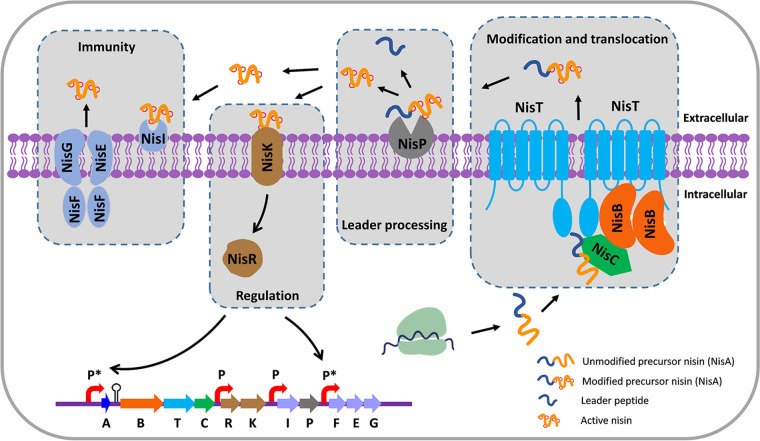
Biosynthesis, regulation, and immunity of nisin in L. lactis. Precursor nisin (NisA) is a ribosomally synthesized peptide with a leader peptide and a core peptide that is then targeted to putative nisin biosynthesis machinery consisting of the dehydratase NisB, the cyclase NisC, and the ABC transporter NisT. NisB converts serine and threonine residues into dehydroalanine and dehydrobutyrine, respectively. NisC catalyzes the addition of a thiol group in cysteine to an N-terminally located dehydroamino acid, resulting in the characteristic lanthionine rings. Subsequently, the transporter NisT exports the fully modified precursor nisin outside the cells, where the serine protease NisP extracellularly removes the leader peptide, releasing active nisin. Immunity is conferred by two different systems, the lipoprotein NisI and the ABC transporter NisFEG, protecting the host from the antimicrobial action of nisin. Extracellularly present nisin binds to NisK, a histidine sensor kinase, which starts the autophorylation of a histidine of NisK. Subsequently, phosphate is transferred to NisR, a transcriptional activator, and therefore the promoters indicated by P* are activated by phosphorylated NisR. The other two promoters (P) are constitutive.

Nisin, the class I lanthipeptide produced by Lactococcus lactis, is one of the best-studied and most commonly used lantibiotics. It contains dehydrated amino acids and five (methyl)lanthionine rings, which are essential for antimicrobial activity (5). The genes for nisin biosynthesis are transcriptionally organized into four operons, i.e., *nisABTC*, *nisIP*, *nisRK*, and *nisFEG*. The ribosomally synthesized precursor nisin (NisA) is encoded by the *nisA* gene. Specific serines and threonines in the core peptide of precursor nisin are intracellularly dehydrated by the dehydratase NisB; subsequently, the dehydrated amino acids are linked by the cyclase NisC, forming thioether ring-like structures (8, 9). Next, the fully modified precursor nisin is exported to the exterior by the ABC transporter NisT, followed by the cleavage of the leader peptide by the extracellularly located serine protease NisP to release active nisin (10, 11, 62). The immunity protein NisI and the ABC transporter NisFEG protect the host from the antimicrobial action of nisin (12, 61). NisRK is the two-component regulatory system that induces the transcription of genes required for nisin biosynthesis and immunity (13). The complete biosynthesis process is schematically summarized in Fig. 1. The successful reconstitution of *in vitro* activity and the high-resolution crystal structures of both NisB and NisC have been reported (14–16). In the dehydration process, a glutamate is transferred from glutamyl-tRNA^Glu^ to specific Ser/Thr side chains within the nisin core peptide, introducing glutamylated intermediates. After glutamate elimination, these Ser/Thr residues are converted to dehydroalanine and dehydrobutyrine, respectively, with absolute stereoselectivity (16). The crystal structure of NisB in complex with its substrate NisA reveals the presence of two separate domains that catalyze the Ser/Thr glutamylation and glutamate elimination steps (15). NisC catalyzes a conjugate addition of a C-terminal cysteine residue with the corresponding dehydrated amino acids to generate five cyclic thioethers, one lanthionine, and four methyllanthionines. The crystal structure of NisC displays two domains: a bowl-forming α-toroid domain of seven α-helices and an SH2-like domain of three β-sheets and two α-helices. In the middle of the formed shallow bowl is the catalytic center with the coordinated zinc ion (14). The directionality of nisin dehydration and ring formation was studied, suggesting an N- to C-terminal direction (17), whereas in the NAI-107 maturation process, MibA is modified by MibB in the opposite C- to N-terminal direction after the first dehydration occurs at the N terminus (18).

NisB is predicted to contain one potential transmembrane segment and is self-assembled into a dimer in solution. NisC is a monomeric zinc-dependent protein. Both NisB and NisC have been shown to be cytoplasmic and membrane associated in preparations of membrane vesicles of L. lactis (19, 20). NisT is an ABC transporter that consists of a transmembrane domain (TMD), which is the translocation channel for precursor nisin, and the nucleotide-binding domain (NBD), which binds and hydrolyses ATP to provide the energy for the translocation of precursor nisin. It is assumed to be a half-transporter and therefore required to form a homodimer to function correctly (3). Interactions between the nisin biosynthesis-associated proteins were studied by coimmunoprecipitation and a yeast two-hybrid system. According to these interactions, the authors suggested that the maturation and secretion of nisin occur at a membrane-associated multimeric lanthionine synthetase complex consisting of the proteins NisB and NisC and the ABC transporter NisT (20). Similarly, the dehydratase SpaB, a NisB homolog responsible for the modification of subtilin, which is a nisin homolog, was proven to be localized at the membrane of Bacillus subtilis (21). Furthermore, a membrane-associated complex composed of the proteins SpaB, SpaC, and SpaT responsible for subtilin maturation and secretion was proposed based on the study of interactions between these components (22). When SpaB and SpaC were heterologously coexpressed in Escherichia coli, SpaB was found to be at least a dimer and to interact with SpaC (23). Additionally, in the biosynthesis process of nukacin ISK-1, a class II lanthipeptide, the enzyme NukM and the ABC transporter NukT were shown to form a membrane-located multimeric protein complex by yeast two-hybrid assays and surface plasmon resonance (SPR) (24). NukM expressed heterologously in Staphylococcus carnosus TM9300 was located at the cytoplasmic membrane even in the absence of NukT. Although some evidence has been reported in support of the existence of a multimeric lanthionine synthetase complex consisting of the enzymes LanB and LanC and the ABC transporter LanT, the direct isolation or assembly *in vitro* of such a complex is still unsuccessful. Currently, only the nisin modification complex has been isolated and characterized. For instance, a pulldown assay demonstrated that the nisin modification enzymes NisB and NisC could be copurified with an engineered His-tagged precursor nisin, and the intermolecular interactions of precursor nisin with its modification enzymes were determined (25). The *in vitro* assembly of the nisin maturation complex was conducted, and the complex was identified to comprise a NisB dimer, a monomer of NisC, and one precursor nisin (26). Despite this, it has been shown that NisB, NisC, and even NisT are able to function independently. The dehydration reactions were still performed by NisB both *in vivo* and *in vitro* when NisC and NisT were absent (16, 27). NisC is still capable of inducing a cyclization reaction even when the other enzyme or the transporter is absent (14). Furthermore, the ABC transporter NisT can transport unmodified and dehydrated precursor nisin in the absence of either NisB or NisC (28). Besides, the nisin modification and secretion machinery NisBTC possesses rather broad substrate specificity, as it can dehydrate, cyclize, and transport peptides, totally unrelated to nisin, when fused to the nisin leader peptide (3). These properties have been widely applied in the development of novel lantibiotics (29–32).

Despite the wealth of current insights into the mechanism of nisin maturation, little information about the assembly process of the nisin biosynthesis machinery is available. Moreover, the subcellular localization of such machinery, i.e., where the modification and secretion of nisin occur in the cell, remains unclear. In this study, we systematically expressed fluorescently labeled proteins of the nisin biosynthesis machinery and therefore for the first time revealed their subcellular localization (and colocalization) in live cells. The formation of the nisin biosynthesis machinery was visualized directly using fluorescence microscopy, suggesting a dynamic balance of the assembly and disassembly of the machinery. Furthermore, we investigated the role that individual proteins play in the assembly of the nisin biosynthesis machinery, revealing that NisB appears to be the driver of the localization of the machinery at the old cell poles. The discovery of the polar localization of the nisin biosynthesis machinery and the recruitment process of NisT will lead to a more comprehensive understanding of the maturation of nisin and help to build an empirically determined model of the assembly of the machinery.

## RESULTS

### A plasmid-based expression system for *nisABTC* yields amounts of active nisin similar to those of a chromosomally integrated expression system.

**FIG 2:**
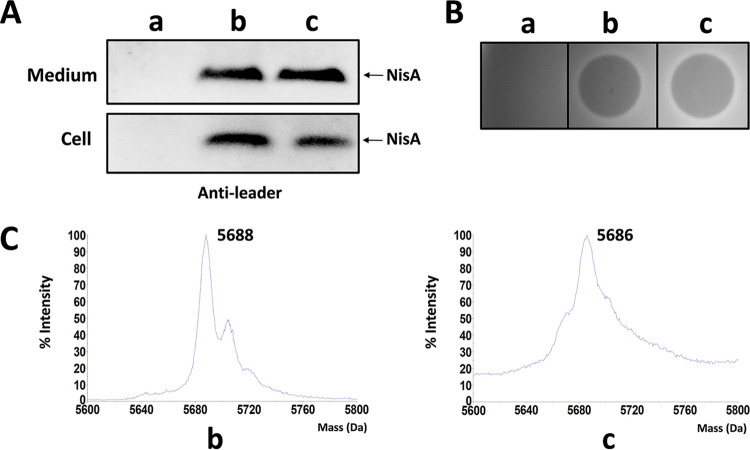
Production of nisin in plasmid-based and chromosomally integrated expression systems. (A) Extracellular and intracellular precursor nisin (NisA) detected by Western blotting using anti-leader peptide antibody. (B) Antimicrobial activity assay. The supernatant of the culture was incubated with the purified protease NisP. The indicator strain is Micrococcus flavus. (C) MALDI-TOF MS data. The predicted mass of fully modified precursor nisin is 5,688 Da. (a) Wild-type strain NZ9000, which does not contain nisin biosynthetic genes; (b) NZ9000/pTLR3-*nisABTC*; (c) NZ9000 *pseudo10*::*nisABTC*; *pseudo 10*, an integration locus in the chromosome of *L. lactis* NZ9000.

A two-plasmid expression system for the efficient production of nisin, involving the pIL plasmid-borne *nisBTC* genes and the pNZ8048 plasmid-borne *nisA* gene, has been widely used in various aspects of research on nisin (3, 25, 33). To investigate the subcellular localization of the precursor nisin (NisA) and the biosynthetic proteins, keeping the original gene operon intact and keeping the expression of proteins close to those under wild-type conditions are very important. Therefore, two new nisin expression systems, a chromosomally expressed system and a plasmid-based expression system, were developed with single or multiple copies of the nisin biosynthetic operon *nisABTC* in the widely used host L. lactis NZ9000 that harbors the *nisRK* genes necessary for induction. The operon *nisABTC* was PCR amplified from the genome of the nisin-producing strain L. lactis NZ9700 (34) and kept complete in the constructed strains so that the transcription of every gene would be directed from the original operon to ensure that the expression of proteins is at a native level. In both expression systems, abundant modified precursor nisin was produced by transcription induction with subinhibitory amounts of the inducer nisin Z (Fig. 2A). After treatment with the purified serine protease NisP, the secreted precursor nisin showed the expected antimicrobial activity against Micrococcus flavus due to the removal of the leader peptide (Fig. 2B). Matrix-assisted laser desorption ionization–time of flight mass spectrometry (MALDI-TOF MS) indicated that the secreted precursor nisin from both systems was fully modified (Fig. 2C). The reason that we made two expression systems was to test the effect of the expression level of the nisin synthetase complex on the nisin yield and the subcellular localization of the nisin biosynthesis machinery. Surprisingly, the amounts of secreted and modified nisin were similar in both production systems, indicating that the plasmid-based system with multiple copies gave no production advantage over the chromosome-based system with single-copy biosynthesis genes. In short, these two expression systems can both be used in the study of the subcellular localization of the nisin biosynthesis machinery.

### Precursor nisin (NisA), NisB, and NisC mainly localize to the cell poles, whereas NisT is distributed in the cytoplasmic membrane circumferentially.

In order to determine the cellular localization of components associated with the nisin biosynthesis machinery, the fluorescent proteins sfGFP (superfolder green fluorescent protein) and mCherry (red fluorescent protein) were used in our study (35–37). In preliminary experiments, the signal from sfGFP or mCherry expressed alone was always diffuse in cells of L. lactis (see Fig. S1A in the supplemental material). The native cytoplasmic protein DnaK labeled by sfGFP did not generate the localization of a polar or membrane pattern, and the signals from the native membrane proteins SecA and RnY labeled by sfGFP were markedly membrane associated (Fig. S1B). Hence, the labeling of fluorescent proteins reflects the distribution of the native proteins in L. lactis.

10.1128/mBio.02825-20.2 FIG S1 Specific localization of sfGFP- or mCherry-labeled protein is not caused by fusing sfGFP or mCherry. (A) The distribution of sfGFP and mCherry was diffuse in the strains NZ9000/pTLR3-*sfgfp* and NZ9000/pTLR3-*mCherry*, respectively. (B) Localization of the native proteins DnaK, SecA, and RnY. The cytoplasmic protein DnaK labeled by sfGFP was diffuse in the strain NZ9000 *dnaK*::*dnaK-sfgfp*. The membrane proteins SecA and RnY labeled by sfGFP were localized in the membrane in the strains NZ9000 *secA*::*secA-sfgfp* and NZ9000 *rnY*::*rnY-sfgfp*, respectively. Download FIG S1, TIF file, 0.8 MB. Copyright © 2020 Chen et al. 2020 Chen et al. This content is distributed under the terms of the Creative Commons Attribution 4.0 International license.

**FIG 3:**
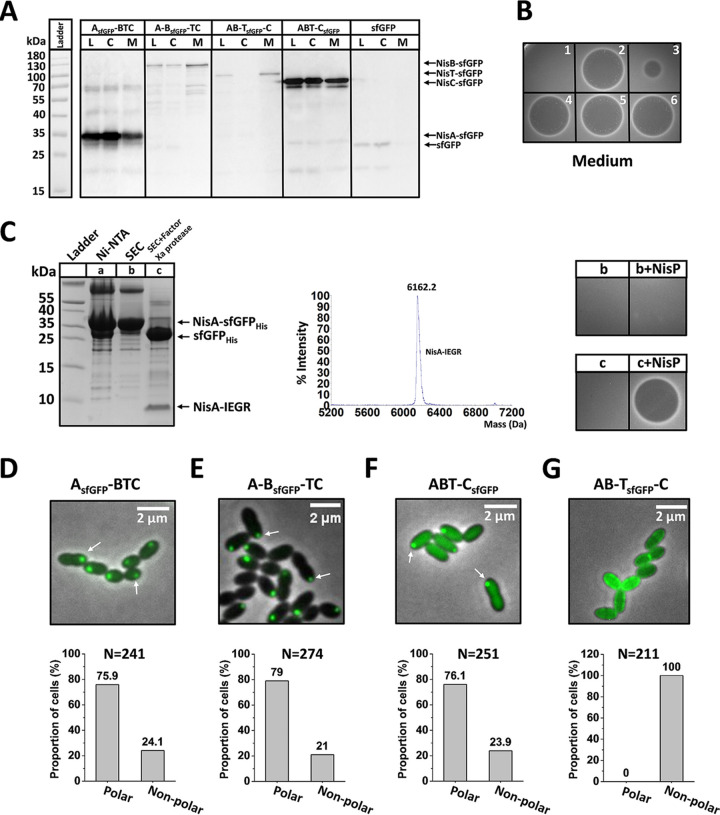
Determination of the subcellular localization of the nisin biosynthesis machinery-associated components using fluorescent protein labeling. (A) Western blot analysis of fusion proteins in the lysate (L), cytosol (C), and membrane (M) fractions. NisA-sfGFP, NisB-sfGFP, NisT-sfGFP, NisC-sfGFP, and sfGFP were determined in different fractions of the strains NZ9000/pTLR3-*nisA_sfgfp_-nisBTC*, NZ9000/pTLR3-*nisA-nisB_sfgfp_-nisTC*, NZ9000/pTLR3-*nisAB*-*nisT_sfgfp_*-*nisC*, NZ9000/pTLR3-*nisABT-nisC_sfgfp_*, and NZ9000/pTLR3-*sfgfp*, respectively. The monoclonal anti-GFP antibody was used. (B) Antimicrobial activity assay. (1) NZ9000/pTLR3, used as a negative control; (2) NZ9000/pTLR3-*nisABTC*; (3) NZ9000/pTLR3-*nisA_sfgfp_-nisBTC*; (4) NZ9000/pTLR3-*nisA-nisB_sfgfp_-nisTC*; (5) NZ9000/pTLR3-*nisAB*-*nisT_sfgfp_*-*nisC*; (6) NZ9000/pTLR3-*nisABT-nisC_sfgfp_*. All the samples were treated with the purified protease NisP. The indicator strain is Micrococcus flavus. (C) Determination of the extent of modification of the NisA portion of the fusion protein NisA-sfGFP. (Left) Purification of NisA-sfGFP_His_ and removal of sfGFP. Ni-NTA, NisA-sfGFP_His_ primarily purified using Ni-NTA agarose; SEC, NisA-sfGFP_His_ further purified by size exclusion chromatography; SEC+Factor Xa protease, sfGFP_His_ removed by incubation with the protease factor Xa. The factor Xa site IEGR was located between NisA and sfGFP_His_. (Middle) MALDI-TOF MS data. The predicted mass of modified NisA-IEGR with 7 dehydrations is 6,161.3 Da. The observed mass is 6,162.2 Da. (Right) Antimicrobial activity assay of NisA-IEGR. The indicator strain is Micrococcus flavus. (D to G) Subcellular localization of sfGFP-labeled proteins and quantification of the proportion of cells with polar fluorescent foci in the strains NZ9000/pTLR3-*nisA_sfgfp_-nisBTC* (D), NZ9000/pTLR3-*nisA-nisB_sfgfp_-nisTC* (E), NZ9000/pTLR3-*nisABT-nisC_sfgfp_* (F), and NZ9000/pTLR3-*nisAB*-*nisT_sfgfp_*-*nisC* (G). N is the number of counted cells from 3 independent experiments. All the above-described analyses were conducted in the plasmid-based expression system.

**FIG 4:**
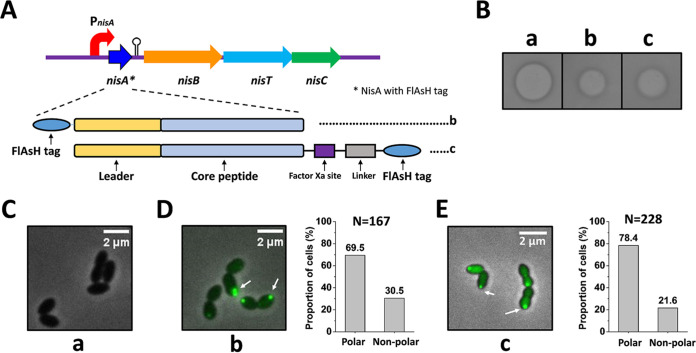
Determination of the localization of precursor nisin using FlAsH labeling. (A) Design of precursor nisin (NisA) tagged by the FlAsH tag. The FlAsH tag was added to the N terminus or the C terminus of precursor nisin, generating MCCPGCC-NisA (peptide b) and NisA-CCPGCC (peptide c). Between precursor nisin and the FlAsH tag, a factor Xa site and a flexible linker were inserted into peptide c. (B) Antimicrobial activity assay. The supernatant of the culture was incubated with purified NisP. The indicator strain is Micrococcus flavus. (C to E) Subcellular localization of precursor nisin with the FlAsH tag and quantification of the proportion of cells with polar fluorescent foci in strains a, b, and c. (a) NZ9000/pTLR3-*nisABTC*, producing precursor nisin without labeling; (b) NZ9000/pTLR3-*_FlAsH_nisA*-*nisBTC*, producing precursor nisin with the FlAsH tag at the N terminus; (c) NZ9000/pTLR3-*nisA_FlAsH_*-*nisBTC*, producing precursor nisin with the FlAsH tag at the C terminus. In panels D and E, percentages were normalized to the total number of bacteria showing a fluorescent signal. N is the number of counted cells from 3 independent experiments. All the above-described analyses were performed using the plasmid-based expression system.

First, we determined the localization of NisA to see if it is specifically localized in bacterial cells. For this, the gene *nisA* was fused, in frame, to a gene encoding sfGFP. A Gly-rich chain was used as a flexible linker joining the peptide and protein to reduce steric interference and thus allow modification of the C-terminal peptide of precursor nisin (38, 39). For convenience, the resulting strains are referred to according to the nisin biosynthesis-associated proteins that they produce, with “A_sfGFP_” being the NisA-sfGFP fusion. Thus, “A_sfGFP_-BTC” represents a strain lacking the native NisA peptide but expressing the NisA-sfGFP fusion as well as NisB, NisT, and NisC. In the plasmid-based expression system (A_sfGFP_-BTC), the genes related to nisin biosynthesis were under the control of the native inducible promoter P*_nisA_*. Western blotting demonstrated that sfGFP was fused to precursor nisin successfully, and the resulting fusion protein NisA-sfGFP was stable in the cells (Fig. 3A). The factor Xa cleavage site IEGR was introduced between NisA and sfGFP, and a His tag was then added to the C terminus of sfGFP, generating NisA-sfGFP_His_. Primary purification of NisA-sfGFP_His_ was performed using Ni-nitrilotriacetic acid (NTA) agarose. The fusion protein was further purified by size exclusion chromatography (SEC). sfGFP_His_ was removed by incubation with the protease factor Xa. The MALDI-TOF result shows that NisA-IEGR was efficiently dehydrated (7 dehydrations). Importantly, after cleavage of the leader peptide by purified NisP, the resulting nisin-IEGR peptide displayed good antimicrobial activity (Fig. 3C). In addition, weak antimicrobial activity of the medium (A_sfGFP_-BTC) was observed, which is likely caused by a small amount of intracellularly degraded sfGFP and subsequently secreted NisA (Fig. 3B). Subsequently, a pulldown assay and Western blotting analysis indicated that a stable complex composed of NisA-sfGFP_His_, NisB, and NisC was isolated (Fig. S2). In short, the above-described data demonstrate that a large attachment of sfGFP fused to the C terminus of NisA neither prevents NisA-sfGFP_His_ binding to NisB and NisC nor affects the modification of the precursor nisin. Fluorescence microscopy of living cells revealed that NisA-sfGFP localized to the cell poles in 75.9% (*n* = 241) of cells with induction with 5 ng/μl nisin Z in the presence of NisB, NisC, and NisT (Fig. 3D). Similarly, in the chromosome-based expression system (A_sfGFP_-BTC), NisA-sfGFP was also polarly localized in cells, with a slightly lower proportion of 65.1% (*n* = 141) (Fig. S3A). To further verify the cellular localization of NisA, we used the tetracysteine-biarsenical system to track its distribution. The short peptide sequence CCXXCC, also named the FlAsH tag, where X is any amino acid other than cysteine, is the smallest and most successful genetically encoded tag for covalent small-fluorophore labeling currently (35). The FlAsH tag CCPGCC was added to either the N terminus or the C terminus of NisA, resulting into MCCPGCC-NisA and NisA-CCPGCC, respectively (Fig. 4A). The supernatants of the cultures containing these two fusions both displayed less antimicrobial activity than that of wild-type precursor nisin after cleaving the leader peptide using purified NisP (Fig. 4B). This suggests that the introduction of the FlAsH tag decreased the efficiency of modification and transportation but did not inhibit the production of the modified peptide. After incubation with FlAsH-EDT_2_, the majority of the bacteria showed detectable fluorescence. Cells with uniform fluorescence and fluorescence concentrated at cell poles were easily identified and counted. The proportion of cells in which FlAsH-stained MCCPGCC-NisA was concentrated at the cell poles was 69.5% (*n* = 167) (Fig. 4D); a similar pattern of polar localization was observed for NisA-CCPGCC in 78.4% (*n* = 228) of cells (Fig. 4E). Hence, the results from FlAsH tag labeling are in line with the sfGFP labeling data, which indicates that precursor nisin in cells has a strong tendency to be concentrated at the poles of L. lactis cells.

10.1128/mBio.02825-20.3 FIG S2 Isolation of the complex comprising NisA-sfGFP_His_, NisB, and NisC via purification of the C-terminally His-tagged NisA-sfGFP fusion. A sample of the strain L. lactis NZ9000/pTLR3-*nisA_sfgfp-His_-nisBTC* was subjected to Ni-NTA purification. Control, strain NZ9000/pTLR3-*nisA_sfgfp_-nisBTC* without a His tag; NisA-sfGFP_His_, fusion protein NisA-sfGFP tagged by a His tag at the C terminus of sfGFP. Elution fractions were analyzed by 8% SDS-PAGE (A) and Western blotting using anti-GFP, anti-leader peptide, anti-NisC, and anti-NisB antibodies (B). Download FIG S2, TIF file, 0.6 MB. Copyright © 2020 Chen et al. 2020 Chen et al. This content is distributed under the terms of the Creative Commons Attribution 4.0 International license.

10.1128/mBio.02825-20.4 FIG S3 Determination of the subcellular localization of nisin biosynthesis machinery-associated components in the chromosome-based expression system. The subcellular localization of sfGFP-labeled proteins and the proportion of cells with polar fluorescent foci were determined in the strains NZ9000 *pseudo10*::*nisA_sfgfp_-nisBTC* (A), NZ9000 *pseudo10*::*nisA-nisB_sfgfp_-nisTC* (B), NZ9000 *pseudo10*::*nisABT-nisC_sfgfp_* (C), and NZ9000 *pseudo10*::*nisAB*-*nisT_sfgfp_*-*nisC* (D). N is the number of counted cells from 3 independent experiments. Download FIG S3, TIF file, 0.6 MB. Copyright © 2020 Chen et al. 2020 Chen et al. This content is distributed under the terms of the Creative Commons Attribution 4.0 International license.

As precursor nisin was mainly localized at the cell poles, we wondered whether other nisin biosynthesis-associated proteins would be similarly localized. Therefore, the fluorescent proteins sfGFP and mCherry were fused to the N and/or C termini of NisB, NisC, and NisT. Similarly, as for NisA-sfGFP, the two proteins were linked through a flexible linker. sfGFP-NisB, mCherry-NisB, sfGFP-NisT, and sfGFP-NisC displayed a weak or almost no fluorescent signal. NisT-mCherry and NisC-mCherry were found to be degraded in the cells. Finally, we screened out the fusion proteins NisB-sfGFP, NisB-mCherry, NisT-sfGFP, mCherry-NisT, NisC-sfGFP, and mCherry-NisC that were functionally active and exhibited good signals in cells to proceed with the following subcellular localization study. In the same way as A_sfGFP_-BTC, “A-B_sfGFP_-TC” is a strain lacking the native NisB protein but expressing the NisB-sfGFP fusion, and “A-B_mCherry_-TC” is a strain lacking the native NisB protein but expressing the NisB-mCherry fusion as well as NisT and NisC. Western blottings showed that sfGFP was fused to NisB, NisC, and NisT successfully (Fig. 3A). Importantly, the fusion proteins were stable, and the fluorescent tag sfGFP was not cleaved off from the tagged proteins, ruling out the possibility of the presence of wild-type enzymes or transporters caused by degradation. We also assessed the ability of NisB-sfGFP, NisC-sfGFP, and NisT-sfGFP to modify or transport precursor nisin by examining the antimicrobial activity of secreted nisin from the culture supernatant after incubation with purified NisP and found that their function was almost indistinguishable from that of the wild-type proteins in the plasmid-based expression system (Fig. 3B). Strikingly, fluorescence microscopy demonstrated that NisB-sfGFP is almost exclusively localized at the cell poles. Quantification of a large number of cells in the plasmid-based expression system (A-B_sfGFP_-TC) indicated that 79% (*n* = 274) of cells had NisB-sfGFP at the polar region, while approximately 21% had nonpolar NisB-sfGFP (Fig. 3E). The observed localization of NisB-sfGFP in the chromosome-based system (A-B_sfGFP_-TC) is consistent with that in the plasmid-based system. Only the proportion (64.4%; *n* = 146) of cells with a polar localization of NisB-sfGFP was slightly lower (Fig. S3B). In addition, we determined the localization of NisB-mCherry and found that it was also primarily localized to the cell poles in the presence of NisA, NisC, and NisT (Fig. S4A). When examined by fluorescence microscopy, NisC-sfGFP was found to be confined to the poles of L. lactis cells in both plasmid-based and chromosome-based expression systems (ABT-C_sfGFP_), with proportions of 76.1% (*n* = 251) and 67.7% (*n* = 186), respectively (Fig. 3F and Fig. S3C), which was in agreement with the data for mCherry-labeled NisC (Fig. S4B), with the coexpression of NisA, NisB, and NisT. These results suggest that NisC is largely localized at the cell poles. Moreover, we identified the spatial distribution of NisT-sfGFP in cells. In both the plasmid- and chromosome-based systems (AB-T_sfGFP_-C), the sfGFP signal was distributed uniformly and circumferentially in a pattern consistent with a membrane localization when NisA, NisB, and NisC were also expressed in cells (Fig. 3G and Fig. S3D). In parallel, we determined the distribution of mCherry-NisT in live cells of the AB-_mCherry_T-C strain (Fig. S4C). It was nearly indistinguishable from that of NisT-sfGFP. These findings imply that the ABC transporter NisT is distributed circumferentially and is not preferentially located at the bacterial poles.

10.1128/mBio.02825-20.5 FIG S4 Determination of the subcellular localization of nisin biosynthesis machinery-associated components labeled by mCherry in the plasmid-based expression system. The localization of mCherry-labeled proteins and the proportion of cells with polar fluorescent foci were determined in the strains NZ9000/pTLR3-*nisA*-*nisB_mCherry_-nisTC* (A), NZ9000/pTLR3-*nisABT*-_mCherry_*nisC* (B), and NZ9000/pTLR3-*nisAB*-*_mCherry_nisT-nisC* (C). N is the number of counted cells from 3 independent experiments. Download FIG S4, TIF file, 0.6 MB. Copyright © 2020 Chen et al. 2020 Chen et al. This content is distributed under the terms of the Creative Commons Attribution 4.0 International license.

From the microscopy images, we noticed that NisA-sfGFP, NisB-sfGFP, and NisC-sfGFP were mainly localized to one pole instead of both poles in single cells. We also tested the effect of expression levels on the subcellular localization of proteins. In plasmid-based systems, the production of fusion proteins was induced with a gradient of concentrations of nisin. The intensity of the signal was enhanced with increasing concentrations of nisin, but the distributions of each fusion protein appeared similar for all concentrations (Fig. S5), which reveals that the localization of NisA, NisB, NisC, and NisT is dose independent. This point is reinforced by the observation of the same distribution of each component in both expression systems. Therefore, we will mainly focus on the plasmid-based system for convenience in the following work.

10.1128/mBio.02825-20.6 FIG S5 Subcellular localization of nisin biosynthesis machinery-associated components is dose independent. The strains NZ9000/pTLR3-*nisA_sfgfp_-nisBTC*, NZ9000/pTLR3-*nisA-nisB_sfgfp_-nisTC*, NZ9000/pTLR3-*nisABT-nisC_sfgfp_*, and NZ9000/pTLR3-*nisAB-nisT_sfgfp_-nisC* were induced by a gradient concentration (0.05, 0.1, 0.3, and 0.5 ng/ml) of nisin Z, and the subcellular localizations of NisA-sfGFP, NisB-sfGFP, NisC-sfGFP, and NisT-sfGFP were identified using fluorescence microscopy, respectively. Download FIG S5, TIF file, 1.9 MB. Copyright © 2020 Chen et al. 2020 Chen et al. This content is distributed under the terms of the Creative Commons Attribution 4.0 International license.

### Nisin modification complex-associated proteins colocalize to the cell poles, with a preference for old poles.

**FIG 5:**
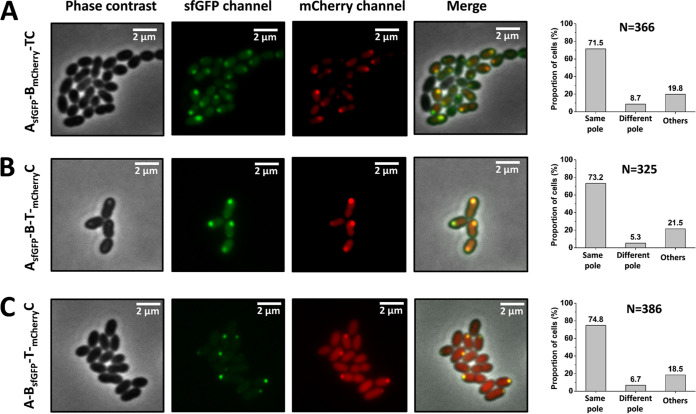
Colocalization of NisA, NisB, and NisC. (A) The fusion proteins NisA-sfGFP and NisB-mCherry were colocalized at the cell poles in the strain NZ9000/pTLR3-*nisA_sfgfp_-nisB_mCherry_-nisTC* in the presence of NisT and NisC. (B) The fusion protein NisA-sfGFP was colocalized with mCherry-NisC to the cell poles in the strain NZ9000/pTLR3-*nisA_sfgfp_-nisBT-_mCherry_nisC* when NisB and NisT were coexpressed. (C) The fusion proteins NisB-sfGFP and mCherry-NisC were colocalized at the same spots of the cell poles of the strain NZ9000/pTLR3-*nisA-nisB_sfgfp_-nisT-_mCherry_nisC* with the coexpression of NisA and NisT. In panels A to C, quantitative analysis of the respective localizations of proteins tagged by sfGFP or mCherry was also performed. Same pole, green foci and red foci colocalized to the same cell poles; Different pole, green foci and red foci localized to different cell poles; Others, no fluorescent focus or weak signal. N is the number of counted cells from 3 independent experiments.

So far, we have presented evidence that the nisin modification machinery-associated proteins localize to the polar region of L. lactis. To show that these proteins assemble at a specific and unique cellular location, we coexpressed proteins fused with different fluorescent proteins. In the A_sfGFP_-B_mCherry_-TC strain, NisA-sfGFP was coexpressed with NisB-mCherry in the presence of NisC and NisT. In 71.5% of the cells (*n* = 366), NisA-sfGFP and NisB-mCherry were found to colocalize at the same pole, as can be observed by merging the different fluorescent images, whereas only 8.7% of cells showed that NisA-sfGFP was localized at poles different from those of NisB-mCherry (Fig. 5A). Similarly, mCherry-NisC was coexpressed with either NisA-sfGFP in the A_sfGFP_-BT-_mCherry_C strain or NisB-sfGFP in the A-B_sfGFP_-T-_mCherry_C strain. NisA-sfGFP and mCherry-NisC were colocalized at the same poles in 73.2% of cells (*n* = 325) when NisB and NisT were coexpressed (Fig. 5B). With the coexpression of NisA and NisT, NisB-sfGFP was colocalized with mCherry-NisC at the same poles in 74.8% of cells (*n* = 386) (Fig. 5C). In both cases, a minority of cells had foci with different fluorescent signals at different cell poles. In summary, these data indicate that NisA, NisB, and NisC colocalize in the polar regions and are functional as a nisin modification complex.

**FIG 6:**
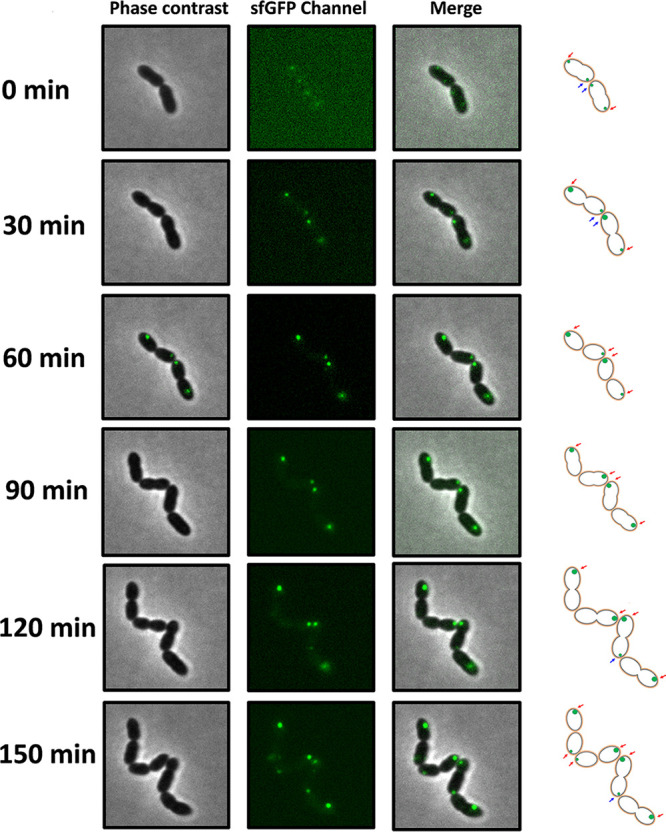
The nisin modification complex represented by NisB-sfGFP was primarily localized to the old pole. Cells from the culture of the strain NZ9000/pTLR3-*nisA*-*nisB_sfgfp_-nisTC* were transferred to a microscope slide with an agarose patch containing growth medium with 5 ng/ml nisin Z as an inducer. Images were captured at 15-min intervals using time-lapse microscopy. The red arrows indicate the appearance of old fluorescent poles. The blue arrows show the appearance of new fluorescent poles.

Although nisin modification-related components were localized to one pole in the majority of cells, a significant proportion (∼10% to 15%) of cells carried NisA-sfGFP, NisB-sfGFP, or NisC-sfGFP at both poles. Notably, one end of the bacterial cell usually was much brighter than the other one (data not shown). Furthermore, some of the cells in which fluorescence was detected at both poles appeared to be longer than the average cells, and a few of them had a constricted middle, revealing that cell division had started. To unambiguously evaluate polar identity, we performed time-lapse microscopy to monitor the distribution variation of NisB-sfGFP, which represents the localization of the nisin modification machinery throughout the cell growth cycle. With a definitive assignment of each resulting “old” and “new” pole, every cell division was observed by capturing images at 15-min intervals. In agreement with the conventional fluorescence microscopy images, the fluorescent signal of the NisB-sfGFP fusion was easily recognized in the time-lapse images and localized to cell poles. In the beginning, fluorescent foci were visualized at both poles in two cells (Fig. 6, red arrows [old poles] and blue arrows [new poles]) (0 and 30 min). After the division of two cells into four, two new poles were treated as old poles. All four fluorescent foci were localized at old poles (60 and 90 min). One new fluorescent focus then appeared at a new pole, previously nonfluorescing, of one cell that was undergoing division (120 min). Afterward, two new fluorescent foci started to be present at old poles in two cells that were newly formed, while other foci were still kept at the original location. In a total of six cells, 6/7 fluorescent foci were present at old cell poles, revealing that the nisin modification complex was mainly localized at the old cell poles (Fig. 6).

### Blocking of nisin secretion allows visualization of the nisin biosynthesis machinery.

Previous research proposed a membrane-associated multimeric lanthionine synthetase complex, which consists of NisA, NisB, NisC, and NisT (3). According to our data described above, NisA, NisB, and NisC are mainly colocalized to the old cell poles, functioning as the nisin modification complex NisABC, while the transporter NisT is uniformly and circumferentially distributed in the cell periphery. This implies that the NisABC complex and NisT are not fully assembled into a complex, which is inconsistent with the putative complex. Western blotting demonstrated that NisB and NisC were not only present in the cytoplasm but also found in the membrane fraction along with NisT (Fig. 3A), which suggests that the modification complex is likely localized at the polar membrane and associated with NisT. Therefore, we hypothesize that the assembly and disassembly of the complex NisABTC occur at the cell poles with a highly dynamic balance that would not lead to abundant accumulation or premature secretion.

**FIG 7:**
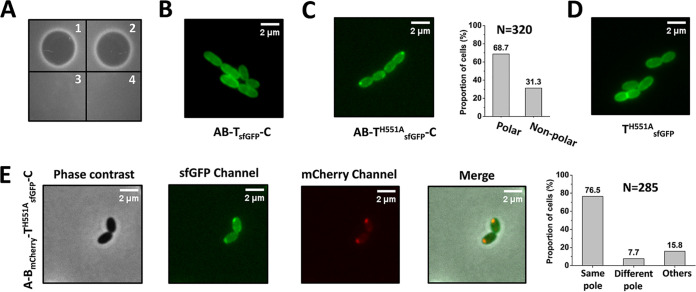
Visualization of the mutant nisin biosynthesis machinery NisBT^H551A^C using fluorescence microscopy. (A) Blocking the secretion of nisin by introducing a mutation, H551A, into NisT. (1) NZ9000/pTLR3-*nisABTC*; (2) NZ9000/pTLR3-*nisAB*-*nisT_sfgfp_*-*nisC*; (3) NZ9000/pTLR3-*nisAB*-*nisT*^H551A^-*nisC*; (4) NZ9000/pTLR3-*nisAB*-*nisT*^H551A^*_sfgfp_*-*nisC*. The supernatant of the culture was incubated with purified NisP. The indicator strain is Micrococcus flavus. (B) Subcellular localization of NisT-sfGFP in the strain NZ9000/pTLR3-*nisAB-nisT_sfgfp_-nisC*. (C) Subcellular localization of NisT^H551A^-sfGFP in the strain NZ9000/pTLR3-*nisAB*-*nisT*^H551A^*_sfgfp_*-*nisC*. NisT^H551A^, mutation H551A introduced into the NBD of the ABC transporter NisT. N is the number of counted cells from 3 independent experiments. (D) Localization of NisT^H551A^-sfGFP in the strain NZ9000/pTLR3-*nisT*^H551A^*_sfgfp_*. (E) Colocalization of NisB-mCherry and NisT^H551A^-sfGFP in the strain NZ9000/pTLR3-*nisA*-*nisB*_mCherry_-*nisT*^H551A^*_sfgfp_*-*nisC* in the presence of NisA and NisC. Same pole, green foci and red foci colocalized to the same cell poles; Different pole, green foci and red foci localized to different cell poles; Others, no fluorescent focus or weak signal. N is the number of counted cells from 3 independent experiments.

To verify this hypothesis, we blocked nisin secretion by introducing a mutation, H551A, into NisT to disturb its function (Fig. 7A). The residue at position 551 is located in the H loop of the NBD, and its mutation to alanine abolished the secretion of nisin (40). Surprisingly, apart from a fluorescent signal that was distributed in a uniform and circumferential pattern, bright foci were seen at the cell poles of the strain AB-T^H551A^_sfGFP_-C, with a cell proportion of 68.7% (*n* = 320), which is different from the distribution of NisT-sfGFP in the strain AB-T_sfGFP_-C (Fig. 7B and C). The formation of bright foci was not caused by the mutation H551A, as no fluorescent focus was detected in the strain T^H551A^_sfGFP_ when NisT^H551A^-sfGFP was expressed alone (Fig. 7D). Because the bright foci were mainly localized at the cell poles, we speculated that their appearance might be relevant to the assembly of the nisin biosynthesis machinery, putting it in a secretion-competent state. Thus, we constructed the strain A-B_mCherry_-T^H551A^_sfGFP_-C and found that NisB-mCherry and NisT^H551A^-sfGFP were colocalized mostly at the cell poles, with the coexpression of NisA and NisC in 76.5% of the cells (*n* = 285) (Fig. 7E). As fluorescently labeled NisB-associated foci represent a fully assembled nisin modification complex, we conclude that the polar bright foci are the assembled complex NisABT^H551A^C. This is the first time that the nisin biosynthesis machinery has been visualized at a specific subcellular location in live cells. We provide direct evidence for the presence of the machinery, although it lost the ability for nisin transportation due to the mutation. More importantly, the hypothesis described above was confirmed. In cells with mutant NisT, intracellular precursor nisin accumulated continually, followed by the formation of more nisin modification machinery at the cell poles. Subsequently, more NisT^H551A^ in the membrane was targeted to cell poles to bind the complex NisABC, leading to the aggregation of the complex NisABT^H551A^C and, therefore, the visualization of bright foci. This is the most likely possibility for the assembly of the mutant nisin biosynthesis machinery. For the wild-type situation, we propose that NisT is recruited henceforth to the poles to secrete precursor nisin as soon as precursor nisin is fully modified and released from NisBC. It is tempting to speculate that NisBC with an unbound leader peptide, which becomes freely accessible, initiates the interaction with “empty” NisT. After the transportation process is finished, NisT disassociates from the machinery and travels back to the peripheral membrane sites.

### NisB plays a central role in the assembly of the nisin biosynthesis machinery.

We speculated that the localization of NisB and other nisin biosynthesis machinery-associated components reflects their incorporation into a macromolecular complex and that in the absence of an intact apparatus or with hampered functionality, these proteins might not localize correctly. Thus, we determined the subcellular localization of enzymes and the transporter in mutant backgrounds of the nisin biosynthesis machinery to study the interactions between different components.

**FIG 8:**
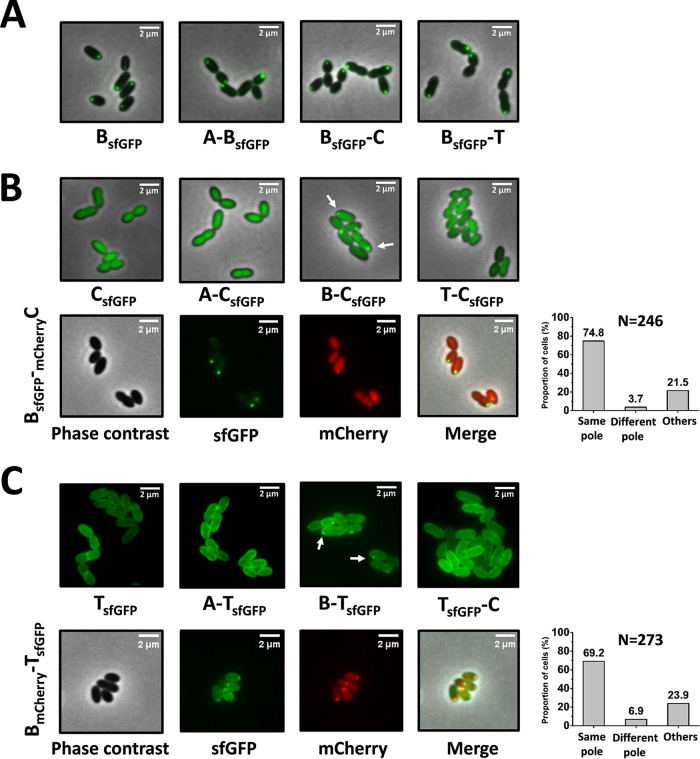
NisC and NisT were targeted to NisB when they were coexpressed with NisB. (A) Effect of coexpression with NisA, NisC, or NisT on the localization of NisB-sfGFP. (B) Effect of coexpression with NisA, NisB, or NisT on the localization of NisC-sfGFP. (C) Effect of coexpression with NisA, NisB, and NisC on the localization of NisT-sfGFP. Same pole, green foci and red foci colocalized to the same cell poles; Different pole, green foci and red foci localized to different cell poles; Others, no fluorescent focus or weak signal. N is the number of counted cells from 3 independent experiments.

When NisB-sfGFP was expressed in bacteria without the presence of NisA, NisC, and NisT, it was primarily localized to the old cell poles, which was identical to its localization in bacteria with intact machinery. Coexpression with NisA, NisC, or NisT did not result in any change of the localization of NisB-sfGFP (Fig. 8A). These results show that the correct localization of NisB is not dependent on other components of the nisin biosynthesis machinery. In strain C_sfGFP_, NisC-sfGFP was expressed in the absence of other components. The fluorescent signal was no longer confined to the cell poles but was distributed diffusely in cells. It must make specific contacts with other components that recruit it to the polar region in bacteria when the intact machinery is present. Next, we coexpressed NisA, NisB, or NisT with NisC-sfGFP. The distribution of NisC-sfGFP was still diffuse when NisA or NisT was present. But in the strain B-C_sfGFP_, NisC-sfGFP showed a polar localization as a result of the coexpression of NisB. Moreover, NisB-sfGFP and mCherry-NisC were colocalized at the same spots in the polar region in 74.8% of cells (*n* = 246) when they were coexpressed in the strain B_sfGFP_-_mCherry_C (Fig. 8B). Besides, in the strain AT-C_sfGFP_ in the absence of NisB, the polar localization of NisC-sfGFP was lost completely, in contrast to that of the strain ABT-C_sfGFP_ with intact machinery (Fig. S6A). These observations suggest that NisC can interact with NisB directly without the presence of the substrate NisA and that the polar localization of NisC is dependent on NisB. In other words, NisB appears to be the driver of the localization of the complex NisABC at the old poles. When only NisT-sfGFP was present in cells, the fluorescent signal was circumferentially distributed in the membrane, without any enhanced bright foci. The introduction of the coexpression of NisA or NisC did not result in any change in NisT-sfGFP localization. However, fluorescent foci appeared at the cell poles when NisT-sfGFP was coexpressed with NisB. Furthermore, the colocalization experiments demonstrate that the fluorescent foci of NisT-sfGFP and NisB-mCherry were colocalized to the cell poles, with a cell proportion of 69.2% (*n* = 273), in the strain B_mCherry_-T_sfGFP_ in the absence of NisA and NisC (Fig. 8C), showing that NisT was targeted to NisB, forming the complex NisBT. Hence, we conclude that NisB can interact with NisT directly. Previously, we showed that aggregation of NisT^H551A^-sfGFP at the cell poles was observed in the strain AB-T^H551A^_sfGFP_-C. However, when NisB was deleted, the bright fluorescent foci no longer appeared in the strain A-T^H551A^_sfGFP_-C (Fig. S6B), implying that the aggregation of NisT^H551A^-sfGFP was dependent on NisB. Taken together, these results show that NisB can interact with NisC and NisT directly and is required for the polar localization of NisC and the visualization of the mutant complex NisABT^H551A^C, revealing that NisB plays a central role in the assembly of the nisin biosynthesis machinery.

10.1128/mBio.02825-20.7 FIG S6 The polar localization of NisC and NisT^H551A^ is dependent on the presence of NisB. (A) The localization of NisC-sfGFP became diffuse in the absence of NisB in the strain NZ9000/pTLR3-*nisAT-nisC_sfgfp_*. (B) There was no appearance of fluorescent NisT^H551A^-sfGFP foci when NisB was not present in the strain NZ9000/pTLR3-*nisA-nisT^H551A^_sfgfp_-nisC*. NisT^H551A^, mutation H551A introduced into the NBD of the ABC transporter NisT. Download FIG S6, TIF file, 0.1 MB. Copyright © 2020 Chen et al. 2020 Chen et al. This content is distributed under the terms of the Creative Commons Attribution 4.0 International license.

### Identification of the domain NisB_750–769_ crucial for NisB polar localization.

**FIG 9:**
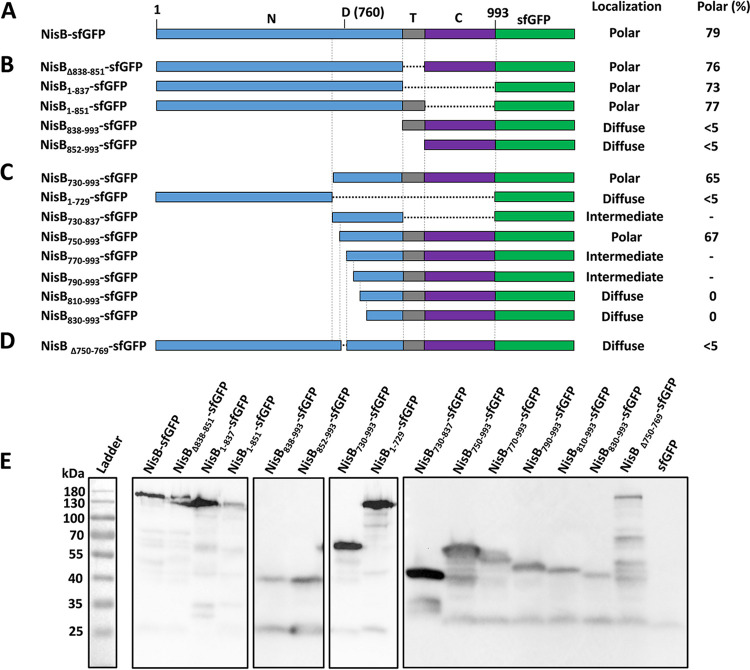
Identification of the domain required for the polar localization of NisB. (A) Native NisB labeled by sfGFP at the C terminus. (B) NisB with deletions based on the predicted transmembrane domain (NisB_838–851_) labeled by sfGFP. (C) NisB with deletions based on the predicted degradation site (residue 760) labeled by sfGFP. (D) NisB with deletion of amino acid residues 750 to 769 labeled by sfGFP. N, N-terminal domain of NisB; D (760), predicted degradation site (residue 760), which is located in the N-terminal domain of NisB; T, predicted transmembrane domain NisB_838–851_; C, C-terminal domain of NisB. (E) Expression of truncated NisB labeled by sfGFP. The monoclonal anti-GFP antibody was used. When the proportion of cells containing polar foci was quantified, the number of cells was higher than 100, and the cells were from 3 independent experiments.

NisB is a 117.5-kDa protein that, according to the UniProt database prediction, contains one potential transmembrane domain, spanning residues 838 to 851 (Fig. 9A). It was reported that a 90-kDa N-terminal NisB fragment, as a result of proteolytic cleavage, could be detected in the cytosol (19, 25). The potential transmembrane domain NisB_838–851_ is located between the N-terminal domain NisB_1–837_ and the C-terminal domain NisB_852–993_. The potential degradation site is predicted to be located near amino acid residue 760, which is included in the N-terminal domain. Based on this information, we generated truncated derivatives of NisB to identify the domains involved in the polar localization of NisB with the coexpression of NisA, NisC, and NisT.

First, a series of truncated NisB proteins labeled C terminally by sfGFP based on the predicted transmembrane domain were constructed (Fig. 9B). Deletions of the predicted transmembrane domain (NisB_Δ838–851_-sfGFP), the C-terminal domain (NisB_1–851_-sfGFP), or both domains (NisB_1–837_-sfGFP) did not affect the polar localization. The deletion of the N-terminal domain (NisB_838–993_-sfGFP) and the fusion of the C-terminal domain with sfGFP (NisB_852–993_-sfGFP) led to diffuse fluorescence. These results show that neither the predicted transmembrane domain nor the C-terminal domain is necessary for the polar localization. Subsequently, a number of sfGFP-labeled truncated NisB variants relevant to the potential degradation site were constructed (Fig. 9C). The N-terminal domain of NisB without the potential degradation site tagged by sfGFP (NisB_1–729_-sfGFP) was diffusely localized, confirming that this part did not contain the residues responsible for the polar localization. The C-terminal part of NisB containing the potential degradation site (NisB_730–993_-sfGFP) was polarly localized, and the short domain with the potential degradation site (NisB_730–837_-sfGFP) showed a mixed pattern, with a diffuse localization and some reinforcement at the pole, which indicated that the domain NisB_730–837_ was required but not sufficient for polar localization. Next, a series of derivatives of NisB_730–993_-sfGFP were generated. NisB_750–993_ labeled by sfGFP was localized to cell poles. A significant decrease in polar localization in cells was observed for both NisB_770–993_-sfGFP and NisB_790–993_-sfGFP, leading to an intermediate pattern between diffuse and polar localizations. NisB_810–993_-sfGFP and NisB_830–993_-sfGFP even completely lost polar localization. The difference in localization between NisB_750–993_-sfGFP and NisB_770–993_-sfGFP suggests that the domain NisB_750–769_ plays a crucial role in targeting NisB to the cell poles. To further test whether amino acid residues 750 to 769 are necessary for the polar localization of intact NisB, we deleted them from full-length NisB. Finally, we found that the deletion of residues 750 to 769 led to a complete loss of polar localization (Fig. 9D). All the constructions described above were proven to be successfully expressed by Western blotting and appeared to be stable (Fig. 9E).

**FIG 10:**
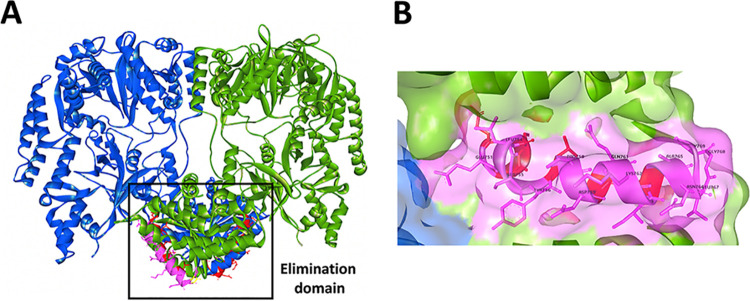
Location of the domain NisB_750–769_ within the crystal structure of NisB (PDB accession no. 4WD9) (15). (A) Overall structure of the NisB homodimer showing the disposition of the domain NisB_750–769_. The NisB homodimer is shown with one monomer in blue and the other monomer in green. The NisB_750–769_ domains are shown in pink and red. The elimination domain is shown in the black square. (B) The majority of residues 750 to 769 are located on the surface of the elimination domain of NisB.

NisB is a tRNA-dependent lantibiotic dehydratase. According to structural information, NisB is composed of an N-terminal glutamylation domain and a C-terminal glutamate elimination domain (15). Within the elimination domain, there is a region, NisB_734–820_, which is similar to the LsrG protein that performs the epimerization of activated quorum-sensing molecules (41). The identified residues 750 to 769 were found to be located in this region, near residues Tyr776, Arg784, and Arg786, which are important in the glutamate elimination step (Fig. 10A). This may imply that there is a certain link between the function and subcellular localization of NisB. Furthermore, we found that the majority of the residues (Glu751, Leu754, Ser755, Tyr756, Pro758, Asp759, Gln761, Lys762, Ile763, Ala765, Asn766, Leu767, Gly768, and, partially, Gly769) within this domain are located on the surface of the elimination domain of NisB (Fig. 10B). It is tempting to speculate that residues 750 to 769 interact with unknown chaperone proteins that assist in the polar localization of NisB. However, the possibility that the loss of polar localization is due to incorrect folding cannot be excluded.

## DISCUSSION

The membrane-associated NisBTC complex for nisin maturation and secretion has been proposed previously, but until now, only indirect evidence to support the existence of such a complex has been reported (20, 22). Knowing the localization of the involved proteins and the underlying dependencies will help in understanding the mechanism of complex assembly. Therefore, in our study, the proteins associated with nisin synthesis were labeled by fluorescent proteins, primarily sfGFP and mCherry, to provide a noninvasive way to visualize where the nisin biosynthesis machinery is localized in bacterial cells. This study addresses fundamental questions concerning the nisin biosynthesis machinery. Do the enzymes and ABC transporter specifically localize within the cells, and if so, do they colocalize with each other, functioning as a complex? If multiple nisin synthesis-associated proteins are employed as reporters, does the absence of other genes affect the localization of the reporters and further define the “order of assembly” of the machinery?

When we performed the labeling approach with fluorescent proteins, while all the proteins can be labeled and produced, their function or interaction with partner proteins may be hampered by the ∼26-kDa tags. To solve this problem, both N- and C-terminal fusions were created, and a polyglycine linker was exploited to join the fluorescent protein with the target protein to avoid steric interference. Finally, we screened out the fusion proteins that were functionally active and exhibited good signals in cells. We expected that fusing sfGFP to precursor nisin could potentially interfere with the posttranslational modifications of the core peptide. However, the NisA portion of the fusion protein NisA-sfGFP_His_ was proven to be efficiently modified and displayed good antimicrobial activity after treatment with NisP. Because precursor nisin is a much smaller peptide than sfGFP, we also performed FlAsH labeling to further verify the distribution of precursor nisin. More importantly, the fluorescent tag was not intracellularly cleaved from the tagged proteins, and the possibility of the specific localization being caused by fusing sfGFP was excluded. Taken together, our results have shown that all the data on the localization associated with the nisin biosynthesis machinery are not artifacts but are reliable.

Our data indicate that the substrate precursor nisin localizes to a single cell pole. The enzymes NisB and NisC, previously reported to form a complex in the presence of precursor nisin, were also proven to be polarly localized. Two-color fluorescence microscopy showed that NisB and NisC colocalized at the same pole as precursor nisin, consistent with the isolation of the nisin modification complex NisABC *in vitro* (25, 26). Furthermore, the images from time-lapse microscopy clearly show that nisin modification complex-associated proteins tend to locate at the “old” but not the “new” cell poles. Based on the hypothesis that precursor nisin, NisB, NisC, and NisT function as a membrane-associated complex (20), we expected that the distribution of NisT would be identical to that of the complex NisABC. However, NisT was uniformly and circumferentially distributed in the cell periphery. Here, we pose the question of whether precursor nisin is secreted at the cell poles or not. We directly visualized the foci of the mutant complex NisABT^H551A^C with the fluorescent tag using fluorescence microscopy when nisin secretion was blocked due to the mutation H551A in the H loop of NisT (40). This is the first study to provide direct evidence of the presence of such a complex *in vivo* and also suggests that the assembly and disassembly of the wild-type NisABTC complex occur at the cell poles with a highly dynamic balance that would not lead to visualized accumulation by fluorescence microscopy. NisT has been reported to be able to transport unmodified precursor nisin when NisB and NisC are absent, but the yield of the peptide is very low (28). In contrast, in the presence of NisB and NisC, NisT can efficiently transport not only fully modified precursor nisin but also other peptides fused to the nisin leader that can be modified. This emphasizes the importance of the association between NisT and NisBC for peptide secretion. Hence, it is tempting to speculate that precursor nisin is transported across the membrane at the cell poles, where NisBC becomes associated with NisT that could be recruited from the initial pool of uniformly and circumferentially distributed NisT in the membrane. In fact, polarized secretion has been reported in many bacteria. For instance, in Agrobacterium tumefaciens, the type IV secretion machinery is known to assemble at the cell poles and determines the polarity of secretion (24, 42). Several structural components of the type II secretion system (T2SS) in Vibrio cholerae localize at one pole, and the secretion of substrate enzymes into the extracellular medium occurs in a polar manner (43). In *Shigella*, the cytoplasmic polar localization directs the secretion of IpaC at the pole and may represent a mandatory step for type III secretion (44). The Esx-1 secretion machine localizes to and is active at the cell poles of mycobacteria (45). In Streptococcus pyogenes, proteins destined for secretion are targeted to a single locus distal to either cell pole that is specialized to contain the Sec translocons (46). Especially, IcsA, a polarly localized autotransporter with an atypical signal peptide, uses the Sec apparatus for secretion, although the Sec apparatus is circumferentially distributed (47). Polar secretion is also advantageous to some bacteria. The secretion of the substrate at poles could result in an increased localized concentration of certain effectors near the cell poles, and such localization might be required to achieve a critical threshold concentration necessary for their activity (48). In our case of L. lactis, the concentration of active nisin outside the cell poles is enhanced continually due to secretion in the polar region of cells so that the threshold concentration is reached faster to activate the action of killing other surrounding Gram-positive bacteria and the immunity procedure via the two-component regulatory system NisRK. Another potential benefit of polar nisin secretion could be to keep secretion away from lipid II. In most bacteria at the poles, there is less peptidoglycan biosynthesis than along the long axis of rod-shaped cells. Notably, the lowest level of cell wall biosynthesis is at the old pole since the new pole (division site) needs peptidoglycan biosynthesis. Hence, it is possible that the reason for the secretion of nisin mainly at old cell poles is to keep the secreted peptides away from lipid II to avoid possible self-killing action.

**FIG 11:**
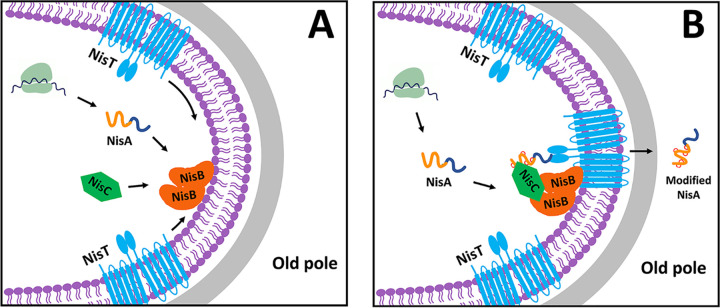
Proposed model of the assembly process and subcellular localization of nisin biosynthesis machinery. (A) NisB plays the role of a “recruiter” in the assembly of the complex NisABTC. NisB is localized to the old cell poles at an early stage. Precursor nisin, NisC, and NisT travel to the poles by binding to NisB via the “diffusion-and-capture” mechanism, generating the nisin biosynthesis machinery. (B) Recruited NisT transports fully modified precursor nisin released from the complex NisBC once the (methyl)lanthionine rings are formed.

A number of mechanisms have been proposed to elucidate how bacteria target proteins to their poles (49, 50). The most common mechanism is a so-called “diffusion-and-capture” mechanism whereby proteins diffuse within the cells, interact with other proteins or protein complexes that are already located at the poles, and subsequently become trapped at that location. Proteins that are independently targeted to the pole have been described as “anchor” or “landmark” proteins (48). Our data demonstrate that NisB localizes to the cell poles without the requirement of the presence of NisA, NisC, and NisT. NisB could also interact with NisC or NisT directly. Importantly, the polar localization of NisC and the appearance of polar NisT^H551A^-sfGFP foci in L. lactis with intact machinery were dependent on the presence of NisB. Hence, it is possible that NisB plays a direct or indirect (via interaction with another protein) role as a polar anchor or landmark protein, and we therefore propose a model for the assembly of the complex NisBTC and the nisin biosynthesis process (Fig. 11). NisB is restricted to the cell poles. When diffuse precursor nisin and NisC encounter NisB in the polar region, they are captured by binding to NisB. The targeting of NisT to the cell poles occurs after membrane insertion: NisT is localized peripherally in the L. lactis membrane, diffuses laterally in the membrane, and is captured by NisBC from the membrane near the cell poles when the complex NisABC is in a translocation-competent state after finishing the modifications. This is similar to the model of the polar localization process of the membrane protein SpoIVFA in B. subtilis (51). Hence, the intact nisin biosynthesis machinery is assembled mainly at the old cell poles (Fig. 11A). In the meantime, fully modified precursor nisin is released from the complex NisBC as soon as the (methyl)lanthionine rings are formed (26) and handed over to the dedicated transporter system NisT, which is associated with NisBC at old pole regions, to be exported outside the cells (Fig. 11B). We hypothesize that the presence of NisBC or the availability of a free leader sequence after the completion of the modification reactions promotes the opening of the channel of NisT so that fully modified precursor nisin can bind. The binding of ATP to the NBD of NisT changes the conformation of NisT dramatically, causing the transportation of fully modified precursor nisin, and meanwhile breaks the interaction with NisB. In the case of NisT^H551A^, since ATP can no longer bind the NBD, NisT is therefore stuck to NisB, leading to its aggregation at the position where NisB is located. Indeed, the polar localization of the mutant machinery NisBT^H551A^C was observed by fluorescence microscopy. This might also indicate a mechanism by which the premature secretion of unfinished precursor nisin is prevented. In the absence of an association with NisBC, the channel of NisT is closed, and it is likely that unmodified nisin has a higher affinity for NisB than for NisT. This mechanism would prevent premature unmodified precursor nisin from being transported. Whether it is a conformational change in NisBC upon the completion of precursor nisin modification or it is the release of the leader from the complex becoming available for the interaction with NisT remains to be established.

The localization of anchor or landmark proteins can take place by several different mechanisms. The poles have several distinctive features, including enhanced negative curvature, an altered lipid composition, and/or a more stable peptidoglycan, and perhaps not surprisingly, bacteria can exploit several of them to efficiently position proteins at the poles (49). For example, the protein DivIVA of Bacillus subtilis preferentially localizes in the most concave regions of the cell via its specific recognition (52). Similarly, the polar localization of the transmembrane E. coli protein ProP is independent of its expression level but correlated with the proportion and polar localization of the anionic phospholipid cardiolipin (53). A second reason for polar localization, exhibited by some chemoreceptor arrays, is based on their ability to self-assemble into large complexes; this self-assembly can occur spontaneously at sites distant from the midcell, likely because of nucleoid exclusion (54). A third mechanism involves the exploitation of the cell division machinery. When bacteria divide, each daughter cell invariably inherits an old pole from its mother and a new pole freshly formed at the site of division. Therefore, a protein stably localized at the division site before cytokinesis could result in localization at a new pole after cell separation (49). B. subtilis DivIVA also localizes to division septa because of the concave membrane curvature and can then recruit other proteins to the site of cell division and future poles (52, 55). In our study, the majority of cells contained only one focus, implying asymmetry of the poles and indicating that localization was not determined simply by membrane curvature. This observation was reinforced by the time-lapse microscopy experiments, which showed that the old pole was the preferred site. During cell division, no focus was observed at the division site (septum); thus, the polar localization of NisB is not correlated with cell division. NisB was shown to be self-assembled into a dimer in previous research (56) and probably localizes to cell poles itself in our study, which is quite similar to the second mechanism. Interestingly, we identified a domain, NisB_750–769_, that is essential for the polar localization of NisB. Highlighting this localization domain on the available NisB structure (Fig. 10) reveals that this domain is located at the surface of the protein. This implies another possibility, that the domain NisB_750–769_ may interact with other potential proteins or molecules, resulting in the observed subcellular localization. Thus, we cannot exclude the possibility that NisB is captured by an unknown actual landmark protein and thereby concentrated at the old cell poles.

In conclusion, we have shown that the nisin biosynthesis machinery is mainly present at the old cell poles. NisB appears to be the driving factor to recruit the other components of the nisin biosynthesis machinery to this location. Interestingly, even a NisT variant that is not able to bind ATP associates with this polar location when NisB is present. Future experiments further examining the secretion site of nisin in the membrane, at old cell poles, and an elucidated structure of NisT will likely reveal key insights about the mechanism of nisin secretion. Finally, future analyses of the interaction between the nisin modification complex NisBC and the ABC transporter NisT will provide more information on key interactions during the assembly process of nisin biosynthesis machinery and tell how precursor nisin is delivered from NisBC to NisT after modification.

## MATERIALS AND METHODS

### Bacterial strains and growth conditions.

Bacterial strains of Escherichia coli and L. lactis and plasmids employed in this study are listed in Table S1 and Table S2 in the supplemental material, respectively. The bacterial strain L. lactis NZ9700 was used as the source of the nisin biosynthetic genes *nisABTC*. Micrococcus flavus was employed as an indicator strain for the detection of modified nisin expression. E. coli DH5α served as a host for cloning and plasmid preparation and was grown in Luria-Bertani (LB) medium at 37°C under aerobic conditions with 100 μg/ml erythromycin. The transformation of E. coli strains was performed according to standard procedures (57). For the purpose of protein expression, L. lactis NZ9000 was grown as a standing culture at 30°C in Difco M17 medium (BD, Franklin Lakes, NJ, USA) with 0.5% (wt/vol) glucose (GM17). Erythromycin was added to a final concentration of 5 μg/ml when required. For all other analyses, chemically defined medium (CDM) (pH 6.8) containing 0.5% (wt/vol) glucose (GCDM) was used to grow L. lactis. To generate chromosomal integration, L. lactis was grown in chemically defined SA medium with the addition of 0.5% (wt/vol) glucose and 20 μg/ml 5-fluoroorotic acid (5-FOA; Sigma-Aldrich, St. Louis, MO, USA) as a sole pyrimidine source, and the procedure was conducted as reported previously (58). When transforming L. lactis NZ9000 with the constructed expression plasmids, a standard protocol for the preparation of competent cells and electroporation was used (59). All chemicals were purchased from Sigma-Aldrich.

10.1128/mBio.02825-20.8 TABLE S1 Strains used in this study. Download Table S1, DOCX file, 0.02 MB. Copyright © 2020 Chen et al. 2020 Chen et al. This content is distributed under the terms of the Creative Commons Attribution 4.0 International license.

10.1128/mBio.02825-20.9 TABLE S2 Plasmids used in this study. Download Table S2, DOCX file, 0.02 MB. Copyright © 2020 Chen et al. 2020 Chen et al. This content is distributed under the terms of the Creative Commons Attribution 4.0 International license.

### Recombinant DNA techniques and oligonucleotides.

The techniques for standard molecular cloning were performed as described previously (57). The GenElute genomic DNA kit (Sigma-Aldrich, St. Louis, MO) was used to isolate genomic DNA of L. lactis. The NucleoSpin Plasmid EasyPure kit (Bioke, Leiden, the Netherlands) and the NucleoSpin gel and PCR cleanup kit (Bioke, Leiden, the Netherlands) were employed to extract plasmids and purify PCR products according to the manufacturers’ instructions. PCRs were conducted with PrimeSTAR Max DNA polymerase (TaKaRa Bio Europe SAS, Saint-Germain-en-Laye, France) according to the manufacturer’s protocol. The obtained PCR products were mixed and treated with Gibson assembly master mix (Bioke, Leiden, the Netherlands), yielding 20-nucleotide overhangs annealing to complementary overhangs. In this procedure, ligase was not needed. The mixtures treated with Gibson assembly master mix were applied to transform E. coli DH5α directly to generate plasmids. Oligonucleotides used in this work were purchased from Biolegio BV (Nijmegen, the Netherlands) and are given in Table S3. Electrocompetent cells of L. lactis were transformed using electroporation with a Bio-Rad Gene Pulser (Bio-Rad Laboratories, Richmond, CA), while for E. coli transformation, competent cells were transformed by heat shock. All nucleotide sequencing was performed at Macrogen Europe (Amsterdam, the Netherlands). The detailed procedures for all plasmid constructions are described in Text S1.

10.1128/mBio.02825-20.1 TEXT S1 Materials and methods. Download Text S1, DOCX file, 0.02 MB. Copyright © 2020 Chen et al. 2020 Chen et al. This content is distributed under the terms of the Creative Commons Attribution 4.0 International license.

10.1128/mBio.02825-20.10 TABLE S3 Oligonucleotides used in this study. Download Table S3, DOCX file, 0.01 MB. Copyright © 2020 Chen et al. 2020 Chen et al. This content is distributed under the terms of the Creative Commons Attribution 4.0 International license.

### Antimicrobial activity assay.

Micrococcus flavus was used as an indicator strain and grown overnight in M17 medium supplemented with 0.5% (wt/vol) glucose (GM17). One hundred microliters of a diluted culture (optical density at 600 nm [OD_600_] of 0.5) was added to 100 ml melted GM17 agar at 45°C and poured into plates. Ten-microliter samples with the addition of 1 μl purified protease NisP (laboratory stock) were dropped onto the plate after the agar was solid. The plates were left overnight at 30°C.

### Mass spectrometry analysis.

One microliter of each sample was spotted, dried, and washed with Milli-Q water on the target. Subsequently, 1 μl of 5 mg/ml α-cyano-4-hydroxycinnamic acid (Sigma-Aldrich) was spotted on top of the samples. An ABI Voyager DE Pro (Applied Biosystems) matrix-assisted laser desorption ionization–time of flight (MALDI-TOF) analyzer operating in linear mode using external calibration was used to obtain mass spectra.

### Protein expression.

L. lactis was grown overnight in GM17 medium with appropriate antibiotics. The culture grown overnight was 5% diluted in fresh GM17 medium and grown at 30°C. When the OD_600_ increased to 0.5, nisin Z (final concentration of 5 ng/ml) was added to induce protein expression. Subsequently, cells were grown for 3 h, the OD_600_ was normalized, and cells were collected by centrifugation and washed once with 50 mM Tris-HCl (pH 7.4). The harvested cells were resuspended in lysis buffer (50 mM NaH_2_PO_4_, 300 mM NaCl, 10 mM imidazole [pH 8.0]) with 10 mg/ml lysozyme and a protease inhibitor and incubated for 60 min at 37°C. A total of 10 mM MgSO_4_ and 100 mg/ml DNase I were added. After incubation for 5 min at 37°C, the suspension was passed three times through a French press machine. Two centrifugation steps at 13,000 × *g* for 10 min at 4°C were performed to remove cell debris, and the cell lysate was obtained. For Ni-NTA purification, a standard procedure was followed and conducted in a cold room (4°C). Five milliliters of lysis buffer was run over the column containing Ni-NTA agarose (50%, 1.0 ml; Qiagen Benelux BV) to equilibrate it. Subsequently, 10 ml of the lysate was flowed through the column material twice to allow His-tagged protein to bind to the Ni-NTA agarose. Next, the column material was washed twice with 10 ml wash buffer (50 mM NaH_2_PO_4_, 300 mM NaCl, 20 mM imidazole [pH 8.0]). Eluents were collected in 5 fractions (0.5 ml each) using elution buffer (50 mM NaH_2_PO_4_, 300 mM NaCl, 250 mM imidazole [pH 8.0]). Protein was further purified by using size exclusion chromatography (SEC) (Superdex-200 column; GE Healthcare). Finally, purified proteins were analyzed by SDS-PAGE and Western blotting.

### Cell fractionation.

The culture of L. lactis grown overnight was 5% diluted in fresh GM17 medium and grown at 30°C. When the OD_600_ was 0.5, nisin Z (final concentration of 5 ng/ml) was added to induce protein expression. Cells were grown for 3 h and harvested by centrifugation. Subsequently, the cytoplasmic and membrane fractions were separated: the cell pellet was washed with 50 mM Tris-HCl (pH 7.4), resuspended in cell lysis buffer, and disrupted by a French press machine. The obtained lysate was centrifuged to remove cell debris. The supernatant was then ultracentrifuged (40,000 × *g* for 1 h at 4°C), and the new supernatant (cytoplasmic fraction) was collected again. The membrane pellet was resuspended in cell lysis buffer and ultracentrifuged again (40,000 × *g* for 30 min at 4°C). Finally, the collected membrane fraction was resuspended in lysis buffer. Bicinchoninic acid (BCA) reagent was used to determine the protein concentrations of all collected fractions, and 30 μg total protein was loaded per lane when SDS-PAGE was performed.

### SDS-PAGE and Western blotting.

The samples for SDS-PAGE were incubated in loading buffer containing 5% (vol/vol) β-mercaptoethanol and boiled for 10 min. SDS-PAGE was performed according to a standard operation manual (57). Western blot analyses were performed using anti-leader peptide, anti-NisB, anti-NisC, and anti-green fluorescent protein (GFP) antibodies.

### Sample preparation for microscopy.

L. lactis cells were grown overnight in M17 medium supplemented with 0.5% (wt/vol) glucose and appropriate antibiotics from freshly isolated colonies on a plate. The culture grown overnight was diluted in GCDM to an OD_600_ of 0.05 and grown at 30°C. When the OD_600_ reached 0.5, nisin Z was added at a final concentration of 5 ng/ml to induce the expression of proteins. Samples containing sfGFP- or mCherry-labeled proteins for microscopic observation were taken at exponential phase and immobilized on agarose (1%, wt/vol, in GCDM)-coated microscope slides to be examined. For FlAsH labeling, the cells containing NisA labeled by a FlAsH tag from liquid cultures were pretreated with 0.65 mM BAL (2,3-dimercapto-1-propanol) for 15 min at 30°C in GCDM to suppress the labeling of endogenous cysteine pairs, followed by washes with GCDM at room temperature. The BAL-treated cells were then exposed to 2.5 μM FlAsH-EDT_2_ for 30 min at 30°C, which was followed by 3 consecutive washes with 0.25 mM BAL in warm GCDM to remove FlAsH-EDT_2_-BAL complexes and unbound or loosely bound FlAsH-EDT_2_. An additional treatment with 0.25 mM BAL in GCDM for 30 min at 30°C was performed to displace FlAsH-EDT_2_ that was tightly bound to nonspecific sites by dithiol-independent hydrophobic interactions (60). After a final wash with warm GCDM only, cells in 1 μl of each sample were immobilized on object slides coated with a flat layer of 1% agarose in GCDM. The fluorescent dyes FlAsH-EDT_2_ and 2,3‐dimercapto‐1‐propanol were both purchased from Sanbio BV.

### Fluorescence microscopy.

All micrographs were captured using a DeltaVision Elite inverted epifluorescence microscope (Applied Precision, GE Healthcare, Issaquah, WA, USA) equipped with a stage holder, a climate chamber, a seven-color combined-set InsightSSI solid-state illumination module, and a scientific complementary metal oxide semiconductor (sCMOS) camera (PCO AG, Kelheim, Germany). A 100× phase-contrast objective (numerical aperture [NA], 1.4; oil immersion, DeltaVision Elite) was used for image capturing, in combination with SoftWorX 3.6.0 software (Applied Precision) to control the microscope setup and to perform single-time-point or time-lapse imaging of cells. The following standard fluorescence filter sets were used: excitation at 475/28 nm and emission at 525/48 nm to visualize sfGFP, excitation at 573.5/33 nm and emission at 607.5/19 nm to visualize mCherry, and excitation at 645/40 nm and emission at 535/45 nm to visualize FlAsH-EDT_2_ bound to CCPGCC-tagged proteins. For time point microscopy, a standard microscope slide was prepared with a layer of solidified agarose (1%, wt/vol, in the appropriate medium), and 1 μl of bacterial cells was loaded onto the agarose. The sample was covered with a standard microscope coverslip for microscopic observations. For time-lapse microscopy, microscope slides were incubated in a temperature-controlled (cube-and-box incubation system; Life Imaging Services) automated microscope (DeltaVision Elite) at 30°C for up to 24 h. Images were captured at 10-min intervals, and the *xyz* positions were stored in the microscope control software SoftWorX.

### Data analysis of microscopy images.

For the colocalization experiments, foci were considered to be colocalized when a minimum of a 50% overlap of the foci occurred. Images were deconvolved with SoftWorX imaging software. Color assignment and overlay images were created using ImageJ and saved as green/red tagged-image file format (TIFF) files. In order to quantify the polar localization of the proteins, images from fluorescence microscopy of L. lactis were analyzed with ImageJ software (National Institutes of Health, Bethesda, MD). All different images were acquired with the same exposure time. Image processing consisted of equivalent adjustments of brightness and contrast on complete images. Gamma and LUT (look-up table) values were not modified and were left as linear on each channel. For statistical analysis and quantification of polar indices, multiple slides from at least 3 independent experiments were used. The analysis was performed on more than 100 individual bacteria (see above and the figures). Cells were scored as having polar foci (1 focus and multiple foci) or nonpolar fluorescence (diffuse fluorescence and no fluorescence), with blind evaluation for quantification. Polar indices were expressed as percentages of polar foci in the fluorescent population. In this study, all the experiments were repeated at least 3 times.
