# Supplementary material for: Subcellular Localization and Assembly Process of the Nisin Biosynthesis Machinery in Lactococcus lactis
Source: mBio. 2020 Nov 10;11(6):e02825-20. doi: 10.1128/mBio.02825-20 (PMC7667030; doi:10.1128/mBio.02825-20)
Supplement: TABLE S1 [file mBio.02825-20-st001.docx]

**Table S1 Strains used in this study**

| **Strains** | **Abbreviation** | **Genotype** | **Source** |
| --- | --- | --- | --- |
| *Lactococcus lactis* NZ9000 | - | MG1363 *pepN::nisRK* | ([1](#_ENREF_1)) |
| *Lactococcus lactis* NZ9700 | - | *nisABTCIPRKFEG*, nisin producer | ([2](#_ENREF_2)) |
| *E. coli* DH5α | - | F^-^∆*lac*U169(Ø80d *lac*Z∆M15) *sup*E44 *hsd*R17 *rec*A1 *gyr*A96 *end*A1 *thi*-1 *rel*A1 | ([3](#_ENREF_3)) |
| *Micrococcus flavus* | - | Indicator strain for nisin | ([4](#_ENREF_4)) |
| SJ01 | - | NZ9000 pTLR3, ery^r^ | This study |
| SJ02 | - | NZ9000 pTLR3-*sfgfp*, ery^r^ | This study |
| SJ03 | - | NZ9000 pTLR3-*mCherry*, ery^r^ | This study |
| LG029 | - | NZ9000 *dnaK::dnaK-sfgfp*, ery^r^ | Lab stock |
| LG027 | - | NZ9000 *secA::secA-sfgfp*, ery^r^ | Lab stock |
| LG030 | - | NZ9000 *rnY::rnY-sfgfp*, ery^r^ | Lab stock |
| SJ04 | ABTC | NZ9000 pTLR3-*nisABTC,* ery^r^ | This study |
| SJ05 | A_sfGFP_-BTC | NZ9000 pTLR3-*nisA_sfgfp_-nisBTC,* ery^r^ | This study |
| SJ06 | A_sfGFP-His_-BTC | NZ9000 pTLR3-*nisA_sfgfp-His_-nisBTC,* ery^r^ | This study |
| SJ07 | A-B_sfGFP_-TC | NZ9000 pTLR3-*nisA-nisB_sfgfp_-nisTC,* ery^r^ | This study |
| SJ08 | A-B_mCherry_-TC | NZ9000 pTLR3-*nisA-nisB_mCherry_-nisTC,* ery^r^ | This study |
| SJ09 | AB-T_sfGFP_-C | NZ9000 pTLR3-*nisAB-nisT_sfgfp_-nisC,* ery^r^ | This study |
| SJ10 | AB-_mCherry_T-C | NZ9000 pTLR3-*nisAB-_mCherry_nisT-nisC,* ery^r^ | This study |
| SJ11 | ABT-C_sfGFP_ | NZ9000 pTLR3-*nisABT-nisC_sfgfp_,* ery^r^ | This study |
| SJ12 | ABT-_mCherry_C | NZ9000 pTLR3-*nisABT-_mCherry_nisC,* ery^r^ | This study |
| SJ13 | - | NZ9000 *pseudo10::nisABTC*, ery^r^ | This study |
| SJ14 | - | NZ9000 *pseudo10::nisA_sfgfp_-nisBTC*, ery^r^ | This study |
| SJ15 | - | NZ9000 *pseudo10::nisA-nisB_sfgfp_-nisTC,* ery^r^ | This study |
| SJ16 | - | NZ9000 *pseudo10::nisAB-nisT_sfgfp_-nisC,* ery^r^ | This study |
| SJ17 | - | NZ9000 *pseudo10::nisABT-nisC_sfgfp_,* ery^r^ | This study |
| SJ18 | A_sfGFP_-B_mCherry_-TC | NZ9000 pTLR3-*nisA_sfgfp_-nisB_mCherry_-nisTC,* ery^r^ | This study |
| SJ19 | A_sfGFP_-BT-_mCherry_C | NZ9000 pTLR3-*nisA_sfgfp_-nisBT-_mCherry_nisC,* ery^r^ | This study |
| SJ20 | A-B_sfGFP_-T-_mCherry_C | NZ9000 pTLR3-*nisA-nisB_sfgfp_-nisT-_mCherry_nisC,* ery^r^ | This study |
| SJ21 | _FlAsH_A-BTC | NZ9000 pTLR3-*_FlAsH_nisA*-*nisBTC*, ery^r^ | This study |
| SJ22 | A_FlAsH_-BTC | NZ9000 pTLR3-*nisA_FlAsH_*-*nisBTC*, ery^r^ | This study |
| SJ23 | AB-T^H551A^_sfGFP_-C | NZ9000 pTLR3-*nisAB*-*nisT*^H551A^*_sfgfp_*-*nisC*, ery^r^ | This study |
| SJ24 | A-T^H551A^_sfGFP_-C | NZ9000 pTLR3-*nisA*-*nisT*^H551A^*_sfgfp_*-*nisC*, ery^r^ | This study |
| SJ25 | T^H551A^_sfGFP_ | NZ9000 pTLR3- *nisT*^H551A^*_sfgfp_*, ery^r^ | This study |
| SJ26 | A-B_mCherry_-T^H551A^_sfGFP_-C | NZ9000 pTLR3-*nisA-nisB_mCherry_*-*nisT*^H551A^*_sfgfp_*-*nisC*, ery^r^ | This study |
| SJ27 | B_sfGFP_ | NZ9000 pTLR3-*nisB_sfgfp_*, ery^r^ | This study |
| SJ28 | A-B_sfGFP_ | NZ9000 pTLR3-*nisA*-*nisB_sfgfp_*, ery^r^ | This study |
| SJ29 | B_sfGFP_-C | NZ9000 pTLR3-*nisB_sfgfp_*-*nisC*, ery^r^ | This study |
| SJ30 | B_sfGFP_-T | NZ9000 pTLR3-*nisB_sfgfp_*-*nisT*, ery^r^ | This study |
| SJ31 | C_sfGFP_ | NZ9000 pTLR3-*nisC_sfgfp_*, ery^r^ | This study |
| SJ32 | A-C_sfGFP_ | NZ9000 pTLR3-*nisA*-*nisC_sfgfp_*, ery^r^ | This study |
| SJ33 | B-C_sfGFP_ | NZ9000 pTLR3-*nisB*-*nisC_sfgfp_*, ery^r^ | This study |
| SJ34 | T-C_sfGFP_ | NZ9000 pTLR3-*nisT*-*nisC_sfgfp_*, ery^r^ | This study |
| SJ35 | B_sfGFP_-_mCherry_C | NZ9000 pTLR3-*nisB_sfgfp_*-*_mCherry_nisC*, ery^r^ | This study |
| SJ36 | AT-C_sfGFP_ | NZ9000 pTLR3-*nisAT*-*nisC_sfgfp_*, ery^r^ | This study |
| SJ37 | T_sfGFP_ | NZ9000 pTLR3-*nisT_sfgfp_*, ery^r^ | This study |
| SJ38 | A-T_sfGFP_ | NZ9000 pTLR3-*nisA*-*nisT_sfgfp_*, ery^r^ | This study |
| SJ39 | B-T_sfGFP_ | NZ9000 pTLR3-*nisB*-*nisT_sfgfp_*, ery^r^ | This study |
| SJ40 | T_sfGFP_-C | NZ9000 pTLR3-*nisT_sfgfp_*-*nisC*, ery^r^ | This study |
| SJ41 | B_mCherry_-T_sfGFP_ | NZ9000 pTLR3-*nisB_mCherry_*-*nisT_sfgfp_*, ery^r^ | This study |
| SJ42 | - | NZ9000 pTLR3-*nisA*-*nisB*^Δ838-851^*_sfgfp_*-*nisTC*, ery^r^ | This study |
| SJ43 | - | NZ9000 pTLR3-*nisA*-*nisB*^1-837^*_sfgfp_*-*nisTC*, ery^r^ | This study |
| SJ44 | - | NZ9000 pTLR3-*nisA*-*nisB*^1-851^*_sfgfp_*-*nisTC*, ery^r^ | This study |
| SJ45 | - | NZ9000 pTLR3-*nisA*-*nisB^838-993^_sfgfp_*-*nisTC*, ery^r^ | This study |
| SJ46 | - | NZ9000 pTLR3-*nisA*-*nisB^852-993^_sfgfp_*-*nisTC*, ery^r^ | This study |
| SJ47 | - | NZ9000 pTLR3-*nisA*-*nisB^730-993^_sfgfp_*-*nisTC*, ery^r^ | This study |
| SJ48 | - | NZ9000 pTLR3-*nisA*-*nisB^1-729^_sfgfp_*-*nisTC*, ery^r^ | This study |
| SJ49 | - | NZ9000 pTLR3-*nisA*-*nisB^730-837^_sfgfp_*-*nisTC*, ery^r^ | This study |
| SJ50 | - | NZ9000 pTLR3-*nisA*-*nisB^750-993^_sfgfp_*-*nisTC*, ery^r^ | This study |
| SJ51 | - | NZ9000 pTLR3-*nisA*-*nisB^770-993sfgfp^*-*nisTC*, ery^r^ | This study |
| SJ52 | - | NZ9000 pTLR3-*nisA*-*nisB^790-993^_sfgfp_*-*nisTC*, ery^r^ | This study |
| SJ53 | - | NZ9000 pTLR3-*nisA*-*nisB^810-993sfgfp^*-*nisTC*, ery^r^ | This study |
| SJ54 | - | NZ9000 pTLR3-*nisA*-*nisB^830-993^_sfgfp_*-*nisTC*, ery^r^ | This study |
| SJ55 | - | NZ9000 pTLR3-*nisA*-*nisB*^Δ750-769^*_sfgfp_*-*nisTC*, ery^r^ | This study |

**Preferences**

1. Kuipers A, de Boef E, Rink R, Fekken S, Kluskens LD, Driessen AJM, Leenhouts K, Kuipers OP, Moll GN. 2004. NisT, the transporter of the lantibiotic nisin, can transport fully modified, dehydrated, and unmodified prenisin and fusions of the leader peptide with non-lantibiotic peptides. Journal of Biological Chemistry 279:22176-22182.

2. de Ruyter PG KO, de Vos WM. 1996. Controlled gene expression systems for *Lactococcus lactis* with the food-grade inducer nisin. Appl Environ Microbiol 62:3662-3667.

3. Chen JQ, Zhao LQ, Fu G, Zhou WJ, Sun YX, Zheng P, Sun JB, Zhang DW. 2016. A novel strategy for protein production using non-classical secretion pathway in *Bacillus subtilis*. Microbial Cell Factories 15:69.

4. van Heel AJ, Mu DD, Montalban-Lopez M, Hendriks D, Kuipers OP. 2013. Designing and Producing Modified, New-to-Nature Peptides with Antimicrobial Activity by Use of a Combination of Various Lantibiotic Modification Enzymes. Acs Synthetic Biology 2:397-404.
