# Supplementary material for: Subcellular Localization and Assembly Process of the Nisin Biosynthesis Machinery in Lactococcus lactis
Source: mBio. 2020 Nov 10;11(6):e02825-20. doi: 10.1128/mBio.02825-20 (PMC7667030; doi:10.1128/mBio.02825-20)
Supplement: TABLE S2 [file mBio.02825-20-st002.docx]

**Table S2 Plasmids used in this study**

| **Plasmids** | **Characteristics** | **Source** |
| --- | --- | --- |
| pTLR3 | *E. coli*/*L. lactis* shuttle vector; empty vector for cloning; nisin inducible promoter P*_nisA_*; ery^r^ | Lab stock |
| pUC57-*sfgfp* | *sfgfp,* encoding sfGFP*,* amp^r^ | Lab stock |
| pSEUDO-P*_usp45_*-*mCherry* | *mCherry,* encoding mCherry, ery^r^ | Lab stock |
| pTLR3-*sfgfp* | *sfgfp*, encoding sfGFP under the control of P*_nisA_*; ery^r^ | This study |
| pTLR3-*mCherry* | *mCherry*, encoding mCherry under the control of P*_nisA_*; ery^r^ | This study |
| pTLR3-*nisABTC* | *nisABTC* under the control of P*_nisA_*; nisin expression; ery^r^ | This study |
| pTLR3-*nisABT*C* | *nisABTC* under the control of P*_nisA_*; nisin expression; *nisT* and *nisC* are separated, but the native RBS for *nisC* is kept intact; ery^r^ |  |
| pTLR3-*nisA_sfgfp_-nisBTC* | P*_nisA_-nisA_sfgfp_-nisBTC;* sfGFP was fused to NisA C-terminally; ery^r^ | This study |
| pTLR3-*nisA_sfgfp-His_-nisBTC* | P*_nisA_-nisA_sfgfp-His_-nisBTC;* The Factor Xa cleavage site IEGR was introduced between NisA and sfGFP; the fusion NisA-sfGFP was tagged by His-tag C-terminally; ery^r^ | This study |
| pTLR3-*nisA-nisB_sfgfp_-nisTC* | P*_nisA_-nisA-nisB_sfgfp_-nisTC;* sfGFP was fused to NisB C-terminally; ery^r^ | This study |
| pTLR3-*nisA-nisB_mCherry_-nisTC* | P*_nisA_-nisA-nisB_mCherry_-nisTC;* mCherry was fused to NisB C-terminally; ery^r^ | This study |
| pTLR3-*nisAB-nisT_sfgfp_-nisC* | P*_nisA_-nisAB-nisT_sfgfp_-nisC*; sfGFP was fused to NisT C-terminally; ery^r^ | This study |
| pTLR3-*nisAB-_mCherry_nisT-nisC* | P*_nisA_-nisAB-_mCherry_nisT-nisC*; mCherry was fused to NisT N-terminally; ery^r^ | This study |
| pTLR3-*nisABT-nisC_sfgfp_* | P*_nisA_-nisABT-nisC_sfgfp_*; sfGFP was fused to NisC C-terminally; ery^r^ | This study |
| pTLR3-*nisABT-_mCherry_nisC* | P*_nisA_-nisABT-_mCherry_nisC*; mCherry was fused to NisC N-terminally; ery^r^ | This study |
| pSEUDO10 | *E. coli*/*L. lactis* shuttle integration vector; P*_nisA_*; ery^r^ | Lab stock |
| pSEUDO10-*nisABTC* | pSEODO10 derivative; *nisABTC* under the control of P*_nisA_*; nisin expression; ery^r^ | This study |
| pSEUDO10- *nisA_sfgfp_-nisBTC* | pSEODO10-*nisABTC* derivative; sfGFP was fused to NisA C-terminally; ery^r^ | This study |
| pSEUDO10-*nisA-nisB_sfgfp_-nisTC* | pSEODO10-*nisABTC* derivative; sfGFP was fused to NisB C-terminally; ery^r^ | This study |
| pSEUDO10-*nisAB-nisT_sfgfp_-nisC* | pSEODO10-*nisABTC* derivative; sfGFP was fused to NisT C-terminally; ery^r^ | This study |
| pSEUDO10-*nisABT-nisC_sfgfp_* | pSEODO10- *nisABTC* derivative; sfGFP was fused to NisC C-terminally; ery^r^ | This study |
| pTLR3-*nisA_sfgfp_-nisB_mCherry_-nisTC* | P*_nisA_*-*nisA_sfgfp_-nisB_mCherry_-nisTC*; sfGFP was fused to NisA C-terminally and mCherry was fused to NisB C-terminally simultaneously; ery^r^ | This study |
| pTLR3-*nisA_sfgfp_-nisBT-_mCherry_nisC* | P*_nisA_*-*nisA_sfgfp_-nisBT-_mCherry_nisC*; sfGFP was fused to NisA C-terminally and mCherry was fused to NisC N-terminally simultaneously; ery^r^ | This study |
| pTLR3-*nisA-nisB_sfgfp_-nisT-_mCherry_nisC* | P*_nisA_*-*nisA-nisB_sfgfp_-nisT-_mCherry_nisC*; sfGFP was fused to NisB C-terminally and mCherry was fused to NisC N-terminally simultaneously; ery^r^ | This study |
| pTLR3-*_FlAsH_nisA*-*nisBTC* | P*_nisA_*-_FlAsH_*nisA*-*nisBTC*; NisA was tagged by FlAsH-tag N-terminally; ery^r^ | This study |
| pTLR3-*nisA_FlAsH_*-*nisBTC* | P*_nisA_*-*nisA_FlAsH_*-*nisBTC*; NisA was tagged by FlAsH- tag C-terminally; ery^r^ | This study |
| pTLR3-*nisAB*-*nisT*^H551A^*_sfgfp_*-*nisC* | P*_nisA_*-*nisAB*-*nisT*^H551A^*_sfgfp_*-*nisC*; the mutation H551A was introduced in NisT C-terminally tagged by sfGFP; ery^r^ | This study |
| pTLR3-*nisA*-*nisT*^H551A^*_sfgfp_*-*nisC* | P*_nisA_*-*nisA*-*nisT*^H551A^*_sfgfp_*-*nisC*; the mutation H551A was introduced in NisT C-terminally tagged by sfGFP; ery^r^ | This study |
| pTLR3-*nisT*^H551A^*_sfgfp_* | P*_nisA_*-*nisT*^H551A^*_sfgfp_*; the mutation H551A was introduced in NisT C-terminally tagged by sfGFP; ery^r^ | This study |
| pTLR3-*nisA-nisB_mCherry_*-*nisT*^H551A^*_sfgfp_*-*nisC* | P*_nisA_*-*nisA-nisB_mCherry_*-*nisT*^H551A^*_sfgfp_*-*nisC*; NisB was C-terminally tagged by mCherry and NisT with the mutation H551A was C-terminally tagged by sfGFP simultaneously; ery^r^ | This study |
| pTLR3-*nisB_sfgfp_* | P*_nisA_*-*nisB_sfgfp_*; NisB was C-terminally tagged by sfGFP; ery^r^ | This study |
| pTLR3-*nisA*-*nisB_sfgfp_* | P*_nisA_*-*nisA*-*nisB_sfgfp_*; NisB was C-terminally tagged by sfGFP; ery^r^ | This study |
| pTLR3-*nisB_sfgfp_*-*nisTC* | P*_nisA_*-*nisB_sfgfp_-nisTC*; NisB was C-terminally tagged by sfGFP; ery^r^ | This study |
| pTLR3-*nisB_sfgfp_*-*nisC* | P*_nisA_*-*nisB_sfgfp_-nisC*; NisB was C-terminally tagged by sfGFP; ery^r^ | This study |
| pTLR3-*nisB_sfgfp_*-*nisT* | P*_nisA_*-*nisB_sfgfp_-nisT*; NisB was C-terminally tagged by sfGFP; ery^r^ | This study |
| pTLR3-*nisC_sfgfp_* | P*_nisA_*-*nisC_sfgfp_*; NisC was C-terminally tagged by sfGFP; ery^r^ | This study |
| pTLR3-*nisA*-*nisC_sfgfp_* | P*_nisA_*-*nisA*-*nisC_sfgfp_*; NisC was C-terminally tagged by sfGFP; ery^r^ | This study |
| pTLR3-*nisB*-*nisC_sfgfp_* | P*_nisA_*-*nisB*-*nisC_sfgfp_*; NisC was C-terminally tagged by sfGFP; ery^r^ | This study |
| pTLR3-*nisAB*-*nisC_sfgfp_* | P*_nisA_*-*nisAB*-*nisC_sfgfp_*; NisC was C-terminally tagged by sfGFP; ery^r^ | This study |
| pTLR3-*nisT*-*nisC_sfgfp_* | P*_nisA_*-*nisT*-*nisC_sfgfp_*; NisC was C-terminally tagged by sfGFP; ery^r^ | This study |
| pTLR3-*nisB_sfgfp_*-*_mCherry_nisC* | P*_nisA_*-*nisB_sfgfp_*-*_mCherry_nisC*; NisB was C-terminally tagged by sfGFP and NisC was N-terminally tagged by mCherry simultaneously; ery^r^ | This study |
| pTLR3-*nisAT*-*nisC_sfgfp_* | P*_nisA_*-*nisAT*-*nisC_sfgfp_*; NisC was C-terminally tagged by sfGFP; ery^r^ | This study |
| pTLR3-*nisT_sfgfp_* | P*_nisA_*-*nisT_sfgfp_*; NisT was C-terminally tagged by sfGFP; ery^r^ | This study |
| pTLR3-*nisA*-*nisT_sfgfp_* | P*_nisA_*-*nisA*-*nisT_sfgfp_*; NisT was C-terminally tagged by sfGFP; ery^r^ | This study |
| pTLR3-*nisB*-*nisT_sfgfp_* | P*_nisA_*-*nisB*-*nisT_sfgfp_*; NisT was C-terminally tagged by sfGFP; ery^r^ | This study |
| pTLR3-*nisAB-nisT_sfgfp_* | P*_nisA_*-*nisAB*-*nisT_sfgfp_*; NisT was C-terminally tagged by sfGFP; ery^r^ | This study |
| pTLR3-*nisT_sfgfp_*-*nisC* | P*_nisA_*-*nisT_sfgfp_*-*nisC*; NisT was C-terminally tagged by sfGFP; ery^r^ | This study |
| pTLR3-*nisB_mCherry_*-*nisT_sfgfp_* | P*_nisA_*-*nisB_mCherry_*-*nisT_sfgfp_*; NisB was C-terminally tagged by mCherry and NisT was C-terminally tagged by sfGFP simultaneously; ery^r^ | This study |
| pTLR3-*nisA*-*nisB*^Δ838-851^*_sfgfp_*-*nisTC* | P*_nisA_*-*nisA*-*nisB*^Δ838-851^*_sfgfp_*-*nisTC*; NisB^Δ838-851^ was C-terminally tagged by sfGFP; ery^r^ | This study |
| pTLR3-*nisA*-*nisB*^1-837^*_sfgfp_*-*nisTC* | P*_nisA_*-*nisA*-*nisB*^1-837^*_sfgfp_*-*nisTC*; NisB^1-837^ was C-terminally tagged by sfGFP; ery^r^ | This study |
| pTLR3-*nisA*-*nisB*^1-851^*_sfgfp_*-*nisTC* | P*_nisA_*-*nisA*-*nisB*^1-851^*_sfgfp_*-*nisTC*; NisB^1-851^ was C-terminally tagged by sfGFP; ery^r^ | This study |
| pTLR3-*nisA*-*nisB*^838-993^*_sfgfp_*-*nisTC* | P*_nisA_*-*nisA*-*nisB*^838-993^*_sfgfp_*-*nisTC*; NisB^838-993^ was C-terminally tagged by sfGFP; ery^r^ | This study |
| pTLR3-*nisA*-*nisB*^852-993^*_sfgfp_*-*nisTC* | P*_nisA_*-*nisA*-*nisB*^852-993^*_sfgfp_*-*nisTC*; NisB^852-993^ was C-terminally tagged by sfGFP; ery^r^ | This study |
| pTLR3-*nisA*-*nisB*^730-993^*_sfgfp_*-*nisTC* | P*_nisA_*-*nisA*-*nisB*^730-993^*_sfgfp_*-*nisTC*; NisB^730-993^ was C-terminally tagged by sfGFP; ery^r^ | This study |
| pTLR3-*nisA*-*nisB*^1-729^*_sfgfp_*-*nisTC* | P*_nisA_*-*nisA*-*nisB*^1-729^*_sfgfp_*-*nisTC*; NisB^1-729^ was C-terminally tagged by sfGFP; ery^r^ | This study |
| pTLR3-*nisA*-*nisB*^730-837^*_sfgfp_*-*nisTC* | P*_nisA_*-*nisA*-*nisB*^730-837^*_sfgfp_*-*nisTC*; NisB^730-837^ was C-terminally tagged by sfGFP; ery^r^ | This study |
| pTLR3-*nisA*-*nisB*^750-993^*_sfgfp_*-*nisTC* | P*_nisA_*-*nisA*-*nisB*^750-993^ *_sfgfp_*-*nisTC*; NisB^750-993^ was C-terminally tagged by sfGFP; ery^r^ | This study |
| pTLR3-*nisA*-*nisB*^770-993^*_sfgfp_*-*nisTC* | P*_nisA_*-*nisA*-*nisB*^770-993^ *_sfgfp_*-*nisTC*; NisB^770-993^ was C-terminally tagged by sfGFP; ery^r^ | This study |
| pTLR3-*nisA*-*nisB*^790-993^*_sfgfp_*-*nisTC* | P*_nisA_*-*nisA*-*nisB*^790-993^ *_sfgfp_*-*nisTC*; NisB^790-993^ was C-terminally tagged by sfGFP; ery^r^ | This study |
| pTLR3-*nisA*-*nisB*^810-993^*_sfgfp_*-*nisTC* | P*_nisA_*-*nisA*-*nisB*^810-993^ *_sfgfp_*-*nisTC*; NisB^810-993^ was C-terminally tagged by sfGFP; ery^r^ | This study |
| pTLR3-*nisA*-*nisB*^830-993^*_sfgfp_*-*nisTC* | P*_nisA_*-*nisA*-*nisB*^830-993^ *_sfgfp_*-*nisTC*; NisB^830-993^ was C-terminally tagged by sfGFP; ery^r^ | This study |
| pTLR3-*nisA*-*nisB*^Δ750-769^*_sfgfp_*-*nisTC* | P*_nisA_*-*nisA*-*nisB*^Δ750-769^*_sfgfp_*-*nisTC*; NisB^Δ750-769^ was C-terminally tagged by sfGFP; ery^r^ | This study |
