# Supplementary material for: Subcellular Localization and Assembly Process of the Nisin Biosynthesis Machinery in Lactococcus lactis
Source: mBio. 2020 Nov 10;11(6):e02825-20. doi: 10.1128/mBio.02825-20 (PMC7667030; doi:10.1128/mBio.02825-20)
Supplement: TEXT S1 [file mBio.02825-20-s0001.docx]

**Text S1 Materials and Methods**

Nucleotide sequences of the primers described below are presented in **Table S3**. Pertinent regions of all plasmids were sequenced to confirm their proper nucleotide sequences. All strains and plasmids that were used or created during this study are given in **Table S1** and **Table S2**, respectively.

**Strain Construction**

The integration plasmids (pSEODO10- *nisABTC*, pSEODO10- *nisA_sfgfp_-nisBTC*, pSEODO10-*nisA-nisB_sfgfp_-nisTC*, pSEODO10-*nisAB-nisT_sfgfp_-nisC* and pSEODO10- *nisABT-nisC_sfgfp_*) were constructed in *E. coli* DH5α and subsequently transferred to *L. lactis* NZ9000. A double-crossover recombination strategy, based on 5-FOA counter-selection, yielded strains SJ13, SJ14, SJ15, SJ16 and SJ17, respectively ([1](#_ENREF_1)). All the resulting strains contain a clean insertion of corresponding genes into the *pseudo10* locus.

All the expression plasmids were derived from pTLR3 and constructed in *E. coli* DH5α. The plasmids were transferred to component cells of *L. lactis* NZ9000 by the approach of electroporation using erythromycin as a selection marker, generating corresponding strains ([2](#_ENREF_2)).

**Plasmid Construction**

pTLR3-*sfgfp* and pTLR3-*mCherry* were created by cloning the DNA fragments *sfgfp* and *mCherry* into the vector pTLR3, respectively, using Gibson Assembly strategy ([3](#_ENREF_3)). The gene *sfgfp* was amplified from pUC57-*sfgfp* using primers PJ01 and PJ02. The gene *mCherry* was amplified using pSEUDO-P*_usp45_*-*mCherry* as template with primer pair PJ03/PJ04. The linear vector was obtained from pTLR3 by PCR using the primers PJ05 and PJ06. In the constructed plasmids, *sfgfp* or *mCherry* was under the control of nisin inducible promoter P*_nisA_*.

To achieve approximately wild-type levels of *nisABTC* expression, plasmids pSEUDO10-*nisABTC* and pTLR3-*nisABTC* was constructed as follows: firstly, the original operon *nisABTC* was amplified from *L. lactis* NZ9700 genomic DNA using primers PJ07 and PJ08. The amplicon was inserted into pSEUDO10 and pTLR3 using Gibson Assembly strategy, resulting into pSEUDO10-*nisABTC* and pTLR3-*nisABTC*, respectively. The linear vector backbones were amplified from the template pSEODO10 and pTLR3 using the primer pairs PJ09/PJ10 and PJ11/PJ12, respectively. These two constructions place *nisABTC* immediately downstream of the P*_nisA_* promoter in the vectors.

To fluorescently label precursor nisin NisA, pTLR3-*nisA_sfgfp_-nisBTC* was created: the *sfgfp* gene was amplified from pUC57-*sfgfp* using primers PJ13/PJ14. The linear vector was obtained by PCR from pTLR3-*nisABTC* using primers PJ15/PJ16. The amplicon was cloned behind the *nisA* gene with the deletion of stop codon into pTLR3-*nisABTC* to give the plasmid pTLR3- *nisA_sfgfp_-nisBTC*. A flexible linker (5’-GGTAGCGGTGGAGGTGGCAGC-3’) was located between *nisA* and *sfgfp* to join them to reduce the interference to each other. To conduct pull-down assay, the plasmid pTLR3-*nisA_sfgfp-His_-nisBTC* was made by cloning a DNA sequence of his-tag (5’-CATCATCACCATCACCAT-3’) into pTLR3-*nisA_sfgfp_-nisBTC* downstream of *sfgfp* with the deletion of stop codon using primers PJ17 and PJ18. FlAsH labelling was also employed to label NisA ([4](#_ENREF_4)). The DNA sequence 5’-TGTTGTCCAGGTTGTTGT-3’ (FlAsH-tag CCPGCC) was inserted downstream of the start codon of *nisA* into pTLR3-*nisABTC* to generate pTLR3-*_FlAsH_nisA-nisBTC* with the primers PJ19 and PJ20. In the same way, pTLR3-*nisA_FlAsH_-nisBTC* was constructed using primers PJ21 and PJ22. The same flexible linker as that of pTLR3- *nisA_sfgfp_-nisBTC* was used to link *nisA* and FlAsH-tag.

Using the same strategy of the construction of pTLR3-*nisA_sfgfp_-nisBTC*, pTLR3-*nisA-nisB_sfgfp_-nisTC* (PJ23/PJ24 and PJ25/PJ26), pTLR3-*nisA-nisB_mCherry_-nisTC* (PJ23/PJ24 and PJ27/PJ28), pTLR3-*nisAB-_mCherry_nisT-nisC* (PJ29/PJ30 and PJ31/PJ32) and pTLR3-*nisABT-nisC_sfgfp_* (PJ33/PJ34 and PJ35/PJ36) were created. Duo to the overlapping region between the 3’-terminus of *nisT* and the 5’-terminus of *nisC* in the original operon *nisABTC* ([5](#_ENREF_5)), a round PCR was performed to separate the genes *nisT* and *nisC* using the primers PJ37 and PJ38, and the intermediate plasmid pTLR3-*nisABT*C* was generated. The native RBS (ribosome-binding site) of *nisC* was kept intact. Subsequently, *sfgfp* was cloned behind *nisT* with the primers PJ39/PJ40 and PJ41/PJ42 and *mCherry* was inserted upstream of *nisC* with the primers PJ43/PJ44 and PJ45/PJ46 into pTLR3-*nisABT*C*, resulting into pTLR3-*nisAB-nisT_sfgfp_-nisC* and pTLR3-*nisABT-_mCherry_nisC*, respectively.

NisA, NisB, NisC or NisT was also labelled on chromosome level by constructing pSEODO10-*nisA_sfgfp_-nisBTC*, pSEODO10-*nisA-nisB_sfgfp_-nisTC*, pSEODO10-*nisAB-nisT_sfgfp_-nisC* and pSEODO10-*nisABT-nisC_sfgfp_*, respectively. The genes fused with *sfgfp* (*nisA_sfgfp_-nisBTC*, *nisA-nisB_sfgfp_-nisTC*, *nisAB-nisT_sfgfp_-nisC* and *nisABT-nisC_sfgfp_*) were amplified from pTLR3-*nisA_sfgfp_-nisBTC*, pTLR3-*nisA-nisB_sfgfp_-nisTC*, pTLR3-*nisAB-nisT_sfgfp_-nisC* and pTLR3-*nisABT-nisC_sfgfp_* using the primers PJ47 and PJ48 and cloned into the vector pSEODO10 to get pSEODO10-*nisA_sfgfp_-nisBTC*, pSEODO10-*nisA-nisB_sfgfp_-nisTC*, pSEODO10-*nisAB-nisT_sfgfp_-nisC* and pSEODO10-*nisABT-nisC_sfgfp_* individually. The linear vector pSEUDO10 was obtained by PCR using primers PJ09 and PJ10.

To use different fluorescent proteins to label the components of the nisin modification complex simultaneously, pTLR3-*nisA_sfgfp_-nisBT-_mCherry_nisC*, pTLR3--*nisA_sfgfp_-nisB_mCherry_-nisTC* and pTLR3-*nisA-nisB_sfgfp_-nisT-_mCherry_nisC* were created by similar approach. For instance, the plasmids pTLR3-*nisA_sfgfp_-nisBT-nisC* and pTLR3-*nisA-nisBT-_mCherry_nisC* were digested with the same enzymes XbaI and SphI repectively. The DNA fragment containing *nisA_sfgfp_* from pTLR3-*nisA_sfgfp_-nisBT-nisC* and the DNA fragment containing *_mCherry_nisC* from pTLR3-*nisA-nisBT-_mCherry_nisC* were collected and finally ligated by T4 ligase, yielding the plasmid pTLR3-*nisA_sfgfp_-nisBT-_mCherry_nisC*.

pTLR3-*nisAB*-*nisT*^H551A^*_sfgfp_*-*nisC* was created by site-directed mutagenesis of pTLR3-*nisAB*-*nisT_sfgfp_*-*nisC* with primers PJ49 and PJ50 ([6](#_ENREF_6)). *nisB* was deleted from pTLR3-*nisAB*-*nisT*^H551A^*_sfgfp_*-*nisC* using primers PJ51 and PJ52 to generate pTLR3-*nisA*-*nisT*^H551A^*_sfgfp_*-*nisC.* To constructed pTLR3-*nisT*^H551A^*_sfgfp_*, two steps of gene deletion in plasmid pTLR3-*nisA*-*nisT*^H551A^*_sfgfp_*-*nisC* was performed: *nisA* was deleted with primers PJ53 and PJ54 and then *nisC* was deleted using primers PJ55 and PJ56*.* To make pTLR3-*nisA-nisB_mCherry_*-*nisT*^H551A^*_sfgfp_*-*nisC*, *mCherry* was cloned downstream of *nisB* with stop codon removal into pTLR3-*nisAB*-*nisT*^H551A^*_sfgfp_*-*nisC*. *mCherry* was amplified by the primers PJ57 and PJ58. The linear vector pTLR3-*nisAB*-*nisT*^H551A^*_sfgfp_*-*nisC* was obtained by PCR using primers PJ59 and PJ60.

pTLR3-*nisA*-*nisB_sfgfp_* was derived from pTLR3-*nisA-nisB_sfgfp_-nisTC* and obtained by deleting *nisTC* using the primers PJ61 and PJ62. Subsequently pTLR3-*nisB_sfgfp_* was made from pTLR3-*nisA*-*nisB_sfgfp_* by removing *nisA* with the primers PJ63 and PJ64. To create pTLR3-*nisB_sfgfp_*-*nisC* and pTLR3-*nisB_sfgfp_*-*nisT*, an intermediate plasmid pTLR3-*nisB_sfgfp_*-*nisTC* was constructed by deleting *nisA* from pTLR3-*nisA*-*nisB_sfgfp_*-*nisTC* using primers PJ63 and PJ64. Then, *nisT* or *nisC* was removed from pTLR3-*nisB_sfgfp_*-*nisTC* to obtain pTLR3-*nisB_sfgfp_*-*nisC* and pTLR3-*nisB_sfgfp_*-*nisT* using the primers PJ65/PJ66 and PJ67/PJ68, respectively.

The gene *nisB* was deleted from the plasmid pTLR3-*nisABT*-*nisC_sfgfp_* with the primers PJ51 and PJ52 leading to pTLR3-*nisAT*-*nisC_sfgfp_*. pTLR3-*nisA*-*nisC_sfgfp_* and pTLR3-*nisT*-*nisC_sfgfp_* were made by deleting *nisT* or *nisA* based on pTLR3-*nisAT*-*nisC_sfgfp_* using the primers PJ69/PJ70 and PJ53/PJ54. pTLR3-*nisC_sfgfp_* was derived from pTLR3-*nisA*-*nisC_sfgfp_* by removing *nisA* using the primers PJ71/PJ72. To create pTLR3-*nisB*-*nisC_sfgfp_*, an intermediate plasmid pTLR3-*nisAB*-*nisC_sfgfp_* was constructed from pTLR3-*nisABT*-*nisC_sfgfp_* using primers PJ73 and PJ74. *nisA* was then deleted from pTLR3-*nisAB*-*nisC_sfgfp_* to obtain pTLR3-*nisB*-*nisC_sfgfp_* using the primers PJ75/PJ76. To create pTLR3-*nisB_sfgfp_*-*_mCherry_nisC*, *mCherry* was inserted upstream of *nisC* into pTLR3-*nisB_sfgfp_*-*nisC*. The gene *mCherry* was amplified from pSEUDO-P*_usp45_*-*mCherry* with the primers PJ77 and PJ78. The linear vector pTLR3-*nisB_sfgfp_*-*nisC* was amplified using the primers PJ79 and PJ80.

To construct the derivatives of pTLR3-*nisAB-nisT_sfgfp_-nisC*, firstly pTLR3-*nisAB-nisT_sfgfp_* was made by deleting *nisC* using the primers PJ55 and PJ56. Therefore, pTLR3-*nisA*-*nisT_sfgfp_* and pTLR3-*nisB*-*nisT_sfgfp_* was created by removing *nisB* or *nisA* from the plasmid pTLR3-*nisAB-nisT_sfgfp_* using the primers PJ51/PJ52 and PJ63/PJ64, respectively. pTLR3-*nisT_sfgfp_* was constructed based on pTLR3-*nisB*-*nisT_sfgfp_* using the primers PJ81 and PJ82. To create pTLR3-*nisB_mCherry_*-*nisT_sfgfp_*, *mCherry* was inserted downstream of *nisB* into pTLR3-*nisB*-*nisT_sfgfp_*. The gene *mCherry* was amplified with the primers PJ83 and PJ84. The linear vector pTLR3-*nisB*-*nisT_sfgfp_* was amplified using the primers PJ85 and PJ86.

All the constructions for the identification of domain responsible for NisB polar localization were created based on pTLR3-*nisA*-*nisB_sfgfp_*-*nisTC* by deleting corresponding parts in the gene *nisB*. The primers PJ87-PJ103 used here were listed in **Table S3**.

**References**

1. Solem C, Defoor E, Jensen PR, Martinussen J. 2008. Plasmid pCS1966, a new selection/counterselection tool for lactic acid bacterium strain construction based on the oroP gene, encoding an orotate transporter from *Lactococcus lactis*. Applied and Environmental Microbiology 74:4772-4775.

2. Holo H, Nes IF. 1989. High-Frequency Transformation, by Electroporation, of *Lactococcus-Lactis* Subsp *Cremoris* Grown with Glycine in Osmotically Stabilized Media. Applied and Environmental Microbiology 55:3119-3123.

3. Gibson DG, Young L, Chuang RY, Venter JC, Hutchison CA, Smith HO. 2009. Enzymatic assembly of DNA molecules up to several hundred kilobases. Nature Methods 6:343-U41.

4. Crivat G, Tokumasu F, Sa JM, Hwang J, Wellems TE. 2011. Tetracysteine-Based Fluorescent Tags to Study Protein Localization and Trafficking in *Plasmodium falciparum*-Infected Erythrocytes. Plos One 6 (8):e22975.

5. Kuipers OP, Beerthuyzen MM, Siezen RJ, Devos WM. 1993. Characterization of the Nisin Gene Cluster NisABTCIPR of *Lactococcus Lactis* - Requirement of Expression of the NisA and NisI Genes for Development of Immunity. European Journal of Biochemistry 216:281-291.

6. Zheng L, Baumann U, Reymond JL. 2004. An efficient one-step site-directed and site-saturation mutagenesis protocol. Nucleic Acids Research 32 (14):e115.
