# Supplementary material for: Subcellular Localization and Assembly Process of the Nisin Biosynthesis Machinery in Lactococcus lactis
Source: mBio. 2020 Nov 10;11(6):e02825-20. doi: 10.1128/mBio.02825-20 (PMC7667030; doi:10.1128/mBio.02825-20)
Supplement: TABLE S3 [file mBio.02825-20-st003.docx]

**Table S3 Oligonucleotides used in this study**

| **Primers** | **Sequence (5’--->3’)** |
| --- | --- |
| PJ01 | ATTATAAGGAGGCACTCAAAATGTCAAAAGGAGAAGAGCTGTTCAC |
| PJ02 | TCATCAAACCTCTGAATTCCTTACTTATAAAGCTCATCCATGCCGTGAG |
| PJ03 | ATTATAAGGAGGCACTCAAAATGAGCAAAGGAGAAGAAGATAACATGG |
| PJ04 | TCATCAAACCTCTGAATTCCTTATTTGTAAAGCTCATCCATTCCGCCAG |
| PJ05 | GGAATTCAGAGGTTTGATGACTTTGACC |
| PJ06 | TTTGAGTGCCTCCTTATAATTTATTTTG |
| PJ07 | ATGAGTACAAAAGATTTTAACTTGGATTTG |
| PJ08 | TCATTTCCTCTTCCCTCCTTTC |
| PJ09 | AAGGAGGGAAGAGGAAATGATAATGAGCACTAGTCAAGGTCG |
| PJ10 | TTAAAATCTTTTGTACTCATTTTGAGTGCCTCCTTATAATTTATTTTG |
| PJ11 | AAGGAGGGAAGAGGAAATGAGGAATTCAGAGGTTTGATGACTTTG |
| PJ12 | TTAAAATCTTTTGTACTCATTTTGAGTGCCTCCTTATAATTTATTTTGTAG |
| PJ13 | GTAGCGGTGGAGGTGGCAGCATGTCAAAAGGAGAAGAGCTGTTCAC |
| PJ14 | AATACTATCCTTTGATTTGGTTACTTATAAAGCTCATCCATGCCGTG |
| PJ15 | CCAAATCAAAGGATAGTATTTTGTTAGTTCAGAC |
| PJ16 | GCTGCCACCTCCACCGCTACCTTTGCTTACGTGAATACTACAATGAC |
| PJ17 | CATCATCACCATCACCATtaaAAGAGAGGAAAAAACATGATAAAAAGTTCATTTAAAG |
| PJ18 | ATGGTGATGGTGATGATGCTTATAAAGCTCATCCATGCCGTGAG |
| PJ19 | ATGTGTTGTCCAGGTTGTTGTAGTACAAAAGATTTTAACTTGGATTTGGTATCTG |
| PJ20 | ACAACAACCTGGACAACACATTTTGAGTGCCTCCTTATAATTTATTTTGTAG |
| PJ21 | TGCTGGTGGTGGAGCTTGTTGTCCAGGTTGTTGTTAACCAAATCAAAGGATAGTATTT  TGTTAGTTC |
| PJ22 | AACAAGCTCCACCACCAGCACCTCCTCCACGTCCTTCAATTTTGCTTACGTGAATACTA  CAATGAC |
| PJ23 | GTAGCGGTGGAGGTGGCAGCATGTCAAAAGGAGAAGAGCTGTTCAC |
| PJ24 | CTTCATCCATCTATTAGTCCTTACTTATAAAGCTCATCCATGCCGTG |
| PJ25 | GGACTAATAGATGGATGAAGTGAAAGAATTCAC |
| PJ26 | GCTGCCACCTCCACCGCTACCTTTCATGTATTCTTCCGAAACAAACAACC |
| PJ27 | GTAGCGGTGGAGGTGGCAGCATGAGCAAAGGAGAAGAAGATAACATGG |
| PJ28 | CTTCATCCATCTATTAGTCCTTATTTGTAAAGCTCATCCATTCCGCCAG |
| PJ29 | CATGAAATGAGGACTAATAGATGAGCAAAGGAGAAGAAGATAACATGG |
| PJ30 | GCTGCCACCTCCACCGCTACCTTTGTAAAGCTCATCCATTCCGCCAGTTG |
| PJ31 | GTAGCGGTGGAGGTGGCAGCATGGATGAAGTGAAAGAATTCACATCAAAAC |
| PJ32 | CTATTAGTCCTCATTTCATGTATTCTTCCGAAAC |
| PJ33 | GTAGCGGTGGAGGTGGCAGCATGTCAAAAGGAGAAGAGCTGTTCAC |
| PJ34 | TCATCAAACCTCTGAATTCCTTACTTATAAAGCTCATCCATGCCGTG |
| PJ35 | GGAATTCAGAGGTTTGATGACTTTGACC |
| PJ36 | GCTGCCACCTCCACCGCTACCTTTCCTCTTCCCTCCTTTCAAAAAATCG |
| PJ37 | GCAATATGAGGATAATGATGAATAAAAAAAATATAAAAAGAAATGTTGAAAAAATT  ATTGCTC |
| PJ38 | CATCATTATCCTCATATTGCTCTGATTATTCATCATTATCCTCATATTGCTCTGAATAAT  AAAG |
| PJ39 | GTAGCGGTGGAGGTGGCAGCATGTCAAAAGGAGAAGAGCTGTTCAC |
| PJ40 | TTACTTATAAAGCTCATCCATGCCGTG |
| PJ41 | TGGATGAGCTTTATAAGTAATCAGAGCAATATGAGGATAATGATG |
| PJ42 | CTGCCACCTCCACCGCTACCTTCATCATTATCCTCATATTGCTCTGAATAATAAAGTTC |
| PJ43 | ATGAGCAAAGGAGAAGAAGATAACATGG |
| PJ44 | CATTTCTTTTTATATTTTTTTTATTCATCATTATCCTCATGCTGCCACCTCCACCGCTAC |
| PJ45 | AAAAAATATAAAAAGAAATGTTGAAAAAATTATTGCTCAATGG |
| PJ46 | TCTTCTTCTCCTTTGCTCATATTGCTCTGATTATTCATCATTATCCTCATATTGCTCTG |
| PJ47 | ATTATAAGGAGGCACTCAAAATGAGTACAAAAGATTTTAACTTGGATTTG |
| PJ48 | ACCTTGACTAGTGCTCATTAGGTCAAAGTCATCAAACCTCTGAATTCC |
| PJ49 | CATTTCTGCTAGTTTGAATGCTGCCAGAAAAGC |
| PJ50 | AAACTAGCAGAAATGAAAATTGAAATATTATTTTCCGAAAGAGC |
| PJ51 | TAGCTTATAAAAATACATGAAATGAGGACTAATAGATGGATGAAG |
| PJ52 | TATTTTTATAAGCTATTTAGCAACCCTAAATAACTTATAAAAATAGG |
| PJ53 | CCAAATCAAAGGATAGTATTTTGTTAGTTCAGAC |
| PJ54 | TATCCTTTGATTTGGTTTGAGTGCCTCCTTATAATTTATTTTGTAGTTCC |
| PJ55 | GGAATTCAGAGGTTTGATGACTTTGACC |
| PJ56 | AAACCTCTGAATTCCTTACTTATAAAGCTCATCCATGCCGTG |
| PJ57 | GTAGCGGTGGAGGTGGCAGCATGAGCAAAGGAGAAGAAGATAACATGG |
| PJ58 | CTTCATCCATCTATTAGTCCTTATTTGTAAAGCTCATCCATTCCGCCAG |
| PJ59 | GGACTAATAGATGGATGAAGTGAAAGAATTCAC |
| PJ60 | GCTGCCACCTCCACCGCTACCTTTCATGTATTCTTCCGAAACAAACAACC |
| PJ61 | GGAATTCAGAGGTTTGATGACTTTGACC |
| PJ62 | AAACCTCTGAATTCCTTACTTATAAAGCTCATCCATGCCGTG |
| PJ63 | CCAAATCAAAGGATAGTATTTTGTTAGTTCAGAC |
| PJ64 | TATCCTTTGATTTGGTTTGAGTGCCTCCTTATAATTTATTTTGTAGTTCC |
| PJ65 | GAGCTTTATAAGTAAATTATTCAGAGCAATATGAGGATAATGATG |
| PJ66 | TTACTTATAAAGCTCATCCATGCCGTG |
| PJ67 | GGAATTCAGAGGTTTGATGACTTTGACC |
| PJ68 | AAACCTCTGAATTCCTTACTTATTCATCATTATCCTCATATTGCTCTG |
| PJ69 | TAGCTTATAAAAATAATTATTCAGAGCAATATGAGGATAATGATG |
| PJ70 | TATTTTTATAAGCTATTTAGCAACCCTAAATAACTTATAAAAATAGG |
| PJ71 | AAGGAGGCACTCAAAATTATTCAGAGCAATATGAGGATAATGATG |
| PJ72 | TTTGAGTGCCTCCTTATAATTTATTTTGTAGTTCC |
| PJ73 | GAAATGAATTATTCAGAGCAATATGAGGATAATGATG |
| PJ74 | TGAATAATTCATTTCATGTATTCTTCCGAAACAAACAACC |
| PJ75 | CCAAATCAAAGGATAGTATTTTGTTAGTTCAGAC |
| PJ76 | TATCCTTTGATTTGGTTTGAGTGCCTCCTTATAATTTATTTTGTAGTTCC |
| PJ77 | ACTTTATTATTCAGAGCAATATGAGCAAAGGAGAAGAAGATAACATGG |
| PJ78 | CATTTCTTTTTATATTTTTTTTATTCATCATTATCCTCATGCTGCCACCTCCACCGCTAC |
| PJ79 | AAAAAATATAAAAAGAAATGTTGAAAAAATTATTGCTCAATGG |
| PJ80 | ATTGCTCTGAATAATAAAGTTTACTTATAAAGCTCATCCATGCCGTG |
| PJ81 | TAGCTTATAAAAATACATGAAATGAGGACTAATAGATGGATGAAG |
| PJ82 | TATTTTTATAAGCTATTTAGCAACCCTAAATAACTTATAAAAATAGG |
| PJ83 | GTAGCGGTGGAGGTGGCAGCATGAGCAAAGGAGAAGAAGATAACATGG |
| PJ84 | CTTCATCCATCTATTAGTCCTTATTTGTAAAGCTCATCCATTCCGCCAG |
| PJ85 | GGACTAATAGATGGATGAAGTGAAAGAATTCAC |
| PJ86 | GCTGCCACCTCCACCGCTACCTTTCATGTATTCTTCCGAAACAAACAACC |
| PJ87 | ATCCGAAACATTGATAAAAGATACTAATAATGATTGGAAAGTCG |
| PJ88 | ATCAATGTTTCGGATAACTCTAAAGTATCAAATCCACC |
| PJ89 | GTAGCGGTGGAGGTGGCAGCATGTCAAAAGGAGAAGAGCTGTTCAC |
| PJ90 | CACCTCCACCGCTACCTTCGGATAACTCTAAAGTATCAAATCCACC |
| PJ91 | CACCTCCACCGCTACCAAGCAAATTAGGAATAATTTTAGAATCGGCACAAAATATTG |
| PJ92 | AGAGGAAAAAACATGGCAATATTTTGTGCCGATTCTAAAATTATTCC |
| PJ93 | CATGTTTTTTCCTCTCTTTATTTTTATAAGCTATTTAGC |
| PJ94 | AGAGGAAAAAACATGACATTGATAAAAGATACTAATAATGATTGGAAAGTCG |
| PJ95 | AGAGGAAAAAACATGGAAAAATTGCCCTTTAACGAGTGG |
| PJ96 | CACCTCCACCGCTACCACGCCGTTCAACCGAAACTCTTTTC |
| PJ97 | AGAGGAAAAAACATGAATGAATTTTTACTGTCGTATCTTCCAGATATTCAG |
| PJ98 | AGAGGAAAAAACATGAATCTATTCTTCCTAAGATATACTGATCCTAAACCAC |
| PJ99 | AGAGGAAAAAACATGTCAGATTTATTTTTAGCTTACGGATCTATTCTTGAAATC |
| PJ100 | AGAGGAAAAAACATGAGGATAATGTCAACTTTTGATATTTCTATTTATGATCAAG |
| PJ101 | AGAGGAAAAAACATGTTTGATACTTTAGAGTTATCCGAAGCAATATTTTGTG |
| PJ102 | TCGTCAAAATCTATTCTTCCTAAGATATACTGATCCTAAACCAC |
| PJ103 | AATAGATTTTGACGATTTATAGAAATGTACAACTTTAGATAAAGC |
